# Association of Muscle Radiodensity and Muscle Mass With Thoracic Aortic Calcification Progression in Dialysis Patients

**DOI:** 10.1002/jcsm.13813

**Published:** 2025-04-17

**Authors:** Xiao‐xu Wang, Jing‐yuan Cao, Yao Wang, Min Li, Shi‐mei Hou, Zhen Zhao, Min Yang, Ping‐ping Ju, Yu‐jia Jiang, Jing‐jie Xiao, Ri‐ring Tang, Hong Liu, Bi‐cheng Liu, Xiao‐liang Zhang, Bin Wang

**Affiliations:** ^1^ Department of Nephrology, Zhongda Hospital Southeast University School of Medicine Nanjing China; ^2^ School of Medicine Southeast University Nanjing China; ^3^ Department of Nephrology, The Affiliated Taizhou People's Hospital of Nanjing Medical University, Taizhou School of Clinical Medicine Nanjing Medical University Taizhou China; ^4^ Department of Nephrology, The Affiliated Hospital of Yangzhou University Yangzhou University Yangzhou China; ^5^ Department of Nephrology, The Third Affiliated Hospital of Soochow University Soochow University Changzhou China; ^6^ Jiangsu Key Laboratory of Molecular and Functional Imaging, Department of Radiology, Zhongda Hospital Southeast University School of Medicine Nanjing China; ^7^ Covenant Health Palliative Institute Edmonton Alberta Canada

**Keywords:** cardiovascular diseases, computed tomography, dialysis, skeletal muscle index, skeletal muscle radiodensity, thoracic aortic calcification

## Abstract

**Background:**

Recent findings have spotlighted sarcopenia as a critical factor exacerbating cardiovascular risk in dialysis patients. However, no studies have investigated the relationship of muscle characteristics with thoracic aortic calcification (TAC). We explored whether skeletal muscle radiodensity (SMD) and skeletal muscle index (SMI) are associated with TAC in dialysis patients.

**Methods:**

In this study, 2517 dialysis patients (between January 2020 and June 2023) from four centres with chest computed tomography (CT) scans were analysed cross‐sectionally. A cohort of 544 initial‐dialysis patients (between January 2014 and December 2020) was followed for TAC progression. Chest CT images were used to assess SMD and SMI at the L1 level, as well as to measure the scores of TAC, including ascending TAC (ATAC), aortic arch calcification (AoAC) and descending TAC (DTAC). Multivariable linear regression models were employed to assess the effects of SMD and SMI on TAC and its progression. Restricted cubic spline was used to assess the potential non‐linear relationships of SMD and SMI with TAC progression.

**Results:**

The mean (SD) age for the cross‐sectional study was 54.8 (14.0) years, with males accounting for 58.2%. Over a mean (SD) follow‐up duration of 3.45 (1.82) years, 85.7% showed TAC progression. Comparing the highest quartile of SMD to the lowest quartile, a significant inverse association was observed with TAC (*β*, −1.08 [−1.42 to −0.75]; *p* < 0.001); similar trends were noted for SMI (*β*, −0.42 [−0.74 to −0.10]; *p* = 0.011). SMD and SMI as continuous variables were also both significantly negatively correlated with TAC. In the longitudinal study, multivariable linear regression models revealed that an increase of 1 SD in SMD resulted in a decrease of 0.10 SD (95% CI, −0.17 to −0.02; *p* = 0.011) in TAC progression, and an increase of 1 SD in SMI resulted in a decrease of 0.12 SD (95% CI, −0.20 to −0.04; *p* = 0.003) in TAC progression. Restricted cubic spline models excluded non‐linear trends for the relationships of SMD and SMI with TAC progression. The associations of SMD and SMI with DTAC were consistent with those observed for TAC, but neither showed a significant association with ATAC.

**Conclusions:**

Higher SMD and higher SMI were significantly associated with lower TAC and its progression in dialysis patients. Improving SMD and SMI could be a new approach for reducing TAC.

## 1 Introduction

Cardiovascular (CV) diseases have been identified as the leading cause of mortality among dialysis patients, responsible for more than half of all deaths [1, 2]. Among various factors contributing to CV diseases, thoracic aortic calcification (TAC) plays a pivotal role, acting as a critical marker of subclinical atherosclerosis [3]. Numerous studies have found that TAC is independently associated with atherosclerotic CV disease (ASCVD), coronary heart disease (CHD) risk, stroke and all‐cause mortality [4, 5, 6, 7]. Therefore, identifying the risk factors for TAC in dialysis patients is of great importance for the prevention and treatment of CV events.

Sarcopenia, characterized by a decline in muscle mass and functionality, has been consistently linked to an elevated susceptibility to CV events [8]. A systematic review and meta‐analyses of dialysis patients reported that the risk for CV events was up to 2.8 times higher in those with sarcopenia compared with patients without sarcopenia [9]. This highlights muscle mass and quality as potential indicators of CV risk, with evidence suggesting a correlation between increased abdominal lean muscle and a reduced TAC prevalence in asymptomatic individuals [10]. Yet, the link between muscle characteristics and TAC in dialysis patients remains unexplored.

Computed tomography (CT) images are widely recognized as a reliable method for evaluating changes in muscle mass and quality [11]. Low skeletal muscle index (SMI, reflecting skeletal muscle mass) and/or skeletal muscle radiodensity (SMD, reflecting skeletal muscle quality) at the third lumbar vertebra (L3) or the first lumbar vertebra (L1) levels based on CT assessment have been shown to be associated with poor prognosis of diseases such as cancer [12], liver cirrhosis [13], acute myeloid leukaemia and septic shock [14, 15]. Our prior multicentre research demonstrated that SMD at L1 level in initial‐dialysis patients could predict the risk of cardiac and all‐cause mortality [16], validating opportunistic chest CT images at L1 as a tool for muscle characteristic assessment in dialysis patients. Additionally, chest CT has proven to be an effective method for the detection and quantification of TAC [17]. This study aimed to investigate how skeletal muscle mass and quality at the L1 level, as assessed through opportunistic chest CT images in dialysis patients, are associated with TAC. Furthermore, it evaluated how these muscle characteristics influence the risk of TAC progression in initial‐dialysis patients. These findings offered a novel insight into the risk factors, as well as potential prevention and treatment approaches for TAC and its progression in dialysis patients.

## 2 Methods

### 2.1 Participants and Ethics

The research encompassed a group of dialysis patients, receiving either haemodialysis (HD) or peritoneal dialysis (PD). A total of 3215 patients in the department of nephrology and blood purification centres were recruited between January 2020 and June 2023 from four clinical centres in China.

Eligible recipients were end‐stage kidney disease patients aged between 18 and 80 who had entered regular dialysis and had undergone chest nonenhanced multislice CT scans, including L1. Patients with acute heart failure or myocardial infarction, malignant tumour, liver failure, inflammatory bowel disease, history of parathyroidectomy, Alzheimer's disease and amputees were excluded (Figure S1). This study utilized clinical data from a previous research project to conduct a longitudinal analysis of patients who initially underwent dialysis treatment from January 2014 to December 2020 across four clinical centres [16]. The primary objective of this follow‐up study was to examine the progression of TAC and evaluate the correlation between muscle characteristics and TAC progression in initial‐dialysis patients. The follow‐up study was completed in June 2023.

Ethical approval for this research was granted by Zhongda Hospital's medical research ethics committee (approval number: 2023ZDSYLL172‐P01), with registration in the Chinese Clinical Trial Registry (registration number: ChiCTR2300075231), and was conducted according to the Declaration of Helsinki. The requirement for signed informed consent was waived. The research adhered to the Strengthening the Reporting of Observational Studies in Epidemiology (STROBE) reporting guideline.

### 2.2 CT Image Analysis for SMI and SMD

Chest CT examinations were performed using the Discovery CT750, Revolution CT and Optima CT 660 (all from GE Healthcare, Milwaukee, WI), SOMATOM Sensation (Siemens Healthineers, Erlangen, Germany) or Ingenuity CT (Philips, Amsterdam, the Netherlands). All CT examinations were performed with the following parameters: 120 kVp; automated dose modulation using automA and smartmA for GE Healthcare machines, CareDose 4D for Siemens Healthineers and DoseRight for Philips; matrix 512 × 512; collimation of 0.625 mm; slice thickness of 5 mm. We utilized a single axial CT scan at the L1 vertebra to analyse the skeletal muscle using 3D Slicer (Version 5.0.3, https://www.slicer.org) [18]. The assessment included skeletal muscle area encompassing the psoas major, erector spinae, quadratus lumborum, transversus abdominis, obliques (both external and internal) and the rectus abdominis. These were evaluated and measured based on attenuation values ranging from −29 to 150 Hounsfield units (HU). SMI was derived by normalizing the muscle area relative to the patient's height squared (cm^2^/m^2^), serving as an indicator of skeletal muscle mass. SMD was assessed using the average radiation attenuation value across the entire muscle area at the L1 level.

### 2.3 Assessment of TAC and Definition of TAC Progression

Chest CT images were imported into the 3D slicer software platform to calculate the whole TAC score and calcification scores of the three segments of thoracic aorta, including ascending thoracic aortic (ATAC), aortic arch (AoAC) and descending thoracic aortic (DTAC). Two experienced radiologists were responsible for tracking the entire length and individual segments of the thoracic aorta, selecting all high‐density plaque images (with a threshold set at ≥ 130 HU) and calculating the volume of calcification. Detailed segmentation rules are available in Method S1. In the cross‐sectional study, based on the mean value of the log‐transformed (TAC + 1), which is 4.42, patients were divided into a low TAC group (< 4.42) and a significant TAC group (≥ 4.42). In the longitudinal cohort, TAC progression was defined and quantified as the annualized absolute rate of TAC progression, calculated using the formula: (TAC score [follow‐up] − TAC score [baseline])/follow‐up duration (years). Patients were categorized into rapid or slow progression groups (including no progression) based on whether their yearly increase in TAC was above or below the cohort's median growth rate.

### 2.4 Potential Covariates

Risk factors and covariates were identified through literature review and clinical expertise [19, 20, 21]. Data collection methods and equipment were uniformly standardized across all four study locations. Baseline characteristics included age, sex, height, weight, systolic blood pressure (SBP), diastolic blood pressure (DBP), smoking history, dialysis modality (HD or PD), dialysis duration, comorbidities (diabetes [DM], hypertension, CHD, hyperlipidaemia and stroke), medication history (vitamin D, calcium supplements, cinacalcet and non–calcium‐based phosphate binders) and laboratory results. Levels of white blood cell count (WBC), haemoglobin (Hb), fasting plasma glucose (FPG), triglycerides (TG), total cholesterol (TC), high‐density lipoprotein cholesterol (HDL‐C), low‐density lipoprotein cholesterol (LDL‐C), albumin (ALB), aspartate transaminase (AST), alanine transaminase (ALT), gamma‐glutamyl transferase (GGT), uric acid (UA), bicarbonate, serum calcium, serum phosphate and intact parathyroid hormone (iPTH) were measured using standard laboratory methods. Corrected serum calcium = total serum calcium (mmol/L) + 0.02 (40 [g/L] − ALB [g/L]) [22]. Cigarette smoking was defined as having smoked over 100 cigarettes in one's lifetime. Body mass index (BMI) = weight (kg)/height squared (m^2^). Definitions of DM, hypertension, CHD, hyperlipidaemia and stroke are detailed in Method S2. Because all participants were Chinese (i.e., Asian), data on race and ethnic categories were not collected.

### 2.5 Statistical Analyses

Continuous variables were presented by mean (SD) or median (IQR). Categorical data were summarized by frequencies (percentages). Due to non‐normal distribution, log‐transformed (TAC + 1) and log‐transformed (TAC progression + 1) were used in the analyses. Given the significant differences in muscle between sexes [23], we analysed SMD and SMI by sex‐specific quartiles. Characteristics were compared using one‐way ANOVA or Kruskal–Wallis for continuous variables and *χ* ^2^ for categorical variables.

In both cross‐sectional and longitudinal analyses, multivariable linear regression models were used to estimate regression coefficients (*β*) and 95% confidence intervals (CIs) for SMD and SMI (as continuous variables and as categorized variables stratified by sex) in relation to TAC and its progression with adjustment for various covariates. Moreover, to further explore the potential non‐linear relationship between SMD and SMI with TAC progression, the independent associations of SMD and SMI with TAC progression were also evaluated using restricted cubic splines. The models' goodness of fit was assessed using adjusted *R* ^2^ and the Akaike information criterion (AIC).

Three models were gradually adjusted for covariates. Model 1 was adjusted for SMI (SMD model), SMD (SMI model), age and sex. Model 2 further incorporated BMI, smoking history, dialysis duration and comorbidities (hypertension and DM). Model 3 further incorporated WBC (log WBC), TG (log (TG + 1)), LDL‐C, iPTH (log iPTH), serum phosphate, corrected serum calcium and vitamin D use. We used the results from Model 3 as our main model. In the longitudinal cohort, dialysis duration was not adjusted for because all participants were initial‐dialysis patients. Additionally, log (baseline calcification score + 1) was included in the fully adjusted model.

In the longitudinal study, we further conducted subgroup analyses stratified by age (< 65 or ≥ 65 years), sex (female or male), BMI (< 25 or ≥ 25 kg/m^2^), smoking (yes or no), DM (yes or no) and hypertension (yes or no). *p* for interactions between these covariates and SMI/SMD were calculated by entering a multiplication term in Model 3. Furthermore, several sensitivity analyses were conducted to test the robustness of our findings. (1) To specifically discern the intrinsic relationship between SMD/SMI and TAC progression, and to mitigate the effect of potential confounding factors, we excluded patients diagnosed with stroke and CHD. (2) Patients were stratified into rapid and slow TAC progressors based on the median annualized rate. The impact of SMD and SMI on TAC progression was evaluated using multivariable logistic regression, providing adjusted odds ratios (aORs) and 95% CIs. (3) To explore the minimum strength of association that any unmeasured confounder would need to fully explain away any association, we calculated the *E* value using VanderWeele and Ding's methodology [24], which is a measure of whether the inclusion of further confounders is likely to lead to the attenuation of results (Method S3).

All analyses were conducted using R Version 4.3.1 (R Foundation for Statistical Computing, Vienna, Austria) or STATA version 16.0 (StataCorp LLC, College Station, TX, USA). Two‐sided *p* values < 0.05 were considered statistically significant.

## 3 Results

### 3.1 Cross‐Sectional Association Between SMD/SMI Separated by Sex and TAC

#### 3.1.1 Baseline Characteristics of the Subjects According to SMD/SMI Quartile

A total of 2517 patients were included in the cross‐sectional study (Figure S1), and the baseline characteristics are shown in Tables 1 and S1. The mean (SD) age was 54.8 (14.0) years, with 1470 males (58.4%) and 1047 females (41.6%), and the mean (SD) BMI was 23.0 (3.8) kg/m^2^. There were 2014 HD patients (80.0%) and 503 PD patients (20.0%), with mean (SD) dialysis duration of 3.5 (4.4) years. Compared with subjects in lower SMD quartiles, those in the highest SMD quartile exhibited a younger demographic and healthier metabolic profiles, characterized by reduced BMI, and a lower prevalence of diabetes, hypertension, CHD, hyperlipidaemia and stroke. They also presented with elevated Hb and ALB levels, along with diminished levels of WBC, FPG, AST and GGT. Compared with patients in lower SMI quartiles, those in the highest quartile were younger with higher BMI, more prevalent diabetes and hypertension, lower Hb and elevated FPG. Additionally, TAC, ATAC, AoAC and DTAC were significantly lower in the highest SMD and SMI quartiles than in the other SMD and SMI quartiles.

**TABLE 1:** Baseline characteristics of total subjects according to the SMD quartile separated by sex.

Characteristic	Overall	SMD quartile separated by sex				*p*
		Q1	Q2	Q3	Q4	
Number	2517	629	630	630	628	
SMD, HU						
Men	35.4 (8.1)	24.8 (4.4)	33.2 (1.6)	38.3 (1.4)	45.4 (3.8)	
Women	30.7 (8.4)	19.9 (3.8)	27.7 (1.8)	33.7 (1.8)	41.4 (4.0)	
Age, years	54.8 (14.0)	65.6 (10.6)	57.9 (11.4)	51.3 (12.0)	44.3 (12.3)	< 0.001
Systolic BP, mm Hg	144.7 (25.4)	143.0 (26.6)	144.8 (26.4)	145.0 (23.9)	146.1 (24.8)	0.179
Diastolic BP, mm Hg	85.2 (15.5)	78.6 (13.8)	83.5 (14.7)	87.3 (14.4)	91.4 (16.2)	< 0.001
BMI, kg/m^2^	23.0 (3.8)	24.0 (4.0)	23.1 (3.6)	22.8 (3.7)	22.1 (3.7)	< 0.001
Smoking history, *n* (%)	318 (12.6%)	82 (13.0%)	89 (14.1%)	82 (13.0%)	65 (10.4%)	0.220
Dialysis duration, years	3.5 (4.4)	3.5 (4.1)	4.0 (4.9)	3.4 (4.3)	3.2 (4.2)	0.016
Dialysis modality, *n* (%)						< 0.001
Haemodialysis	2014 (80.0%)	572 (90.9%)	522 (82.9%)	484 (76.8%)	436 (69.4%)	
Peritoneal dialysis	503 (20.0%)	57 (9.1%)	108 (17.1%)	146 (23.2%)	192 (30.6%)	
Diabetes, *n* (%)	772 (30.7%)	309 (49.1%)	217 (34.4%)	160 (25.4%)	86 (13.7%)	< 0.001
Hypertension, *n* (%)	2167 (86.1%)	563 (89.5%)	550 (87.3%)	544 (86.3%)	510 (81.2%)	< 0.001
Coronary heart disease, *n* (%)	310 (12.3%)	152 (24.2%)	83 (13.2%)	50 (7.9%)	25 (4.0%)	< 0.001
Hyperlipidaemia, *n* (%)	434 (17.2%)	158 (25.1%)	121 (19.2%)	103 (16.3%)	52 (8.3%)	< 0.001
Stroke, *n* (%)	364 (14.5%)	160 (25.4%)	97 (15.4%)	75 (11.9%)	32 (5.1%)	< 0.001
Medication history, *n* (%)						
Vitamin D	1252 (49.7%)	278 (44.2%)	305 (48.4%)	329 (52.2%)	340 (54.1%)	0.002
Calcium supplements	680 (27.0%)	193 (30.7%)	167 (26.5%)	174 (27.6%)	146 (23.2%)	0.029
Cinacalcet	365 (14.5%)	80 (12.7%)	94 (14.9%)	102 (16.2%)	89 (14.2%)	0.361
Non–calcium‐containing phosphate binders	1083 (43.0%)	208 (33.1%)	283 (44.9%)	281 (44.6%)	311 (49.5%)	< 0.001
Laboratory results						
WBC, * 10^9^/L	6.2 (4.9–7.8)	6.5 (5.1–8.3)	6.1 (4.9–7.8)	6.1 (4.8–7.6)	6.0 (4.8–7.5)	0.001
Haemoglobin, g/L	97.5 (22.4)	95.0 (23.3)	96.7 (21.9)	98.6 (22.4)	99.6 (21.6)	0.001
Albumin, g/L	34.9 (5.6)	34.0 (5.7)	34.5 (5.6)	35.4 (5.7)	35.8 (5.3)	< 0.001
FPG, μmol/L	5.2 (4.4–6.9)	5.8 (4.6–8.7)	5.3 (4.4–7.0)	5.0 (4.3–6.5)	4.8 (4.2–6.0)	< 0.001
Uric acid, μmol/L	393.9 (132.1)	381.2 (135.8)	392.7 (126.2)	395.2 (129.5)	406.6 (136.0)	0.008
Triglycerides, μmol/L	1.4 (1.0–2.0)	1.4 (0.9–2.0)	1.4 (1.0–2.1)	1.4 (1.0–2.0)	1.4 (1.0–2.0)	0.919
Total cholesterol, μmol/L	3.8 (1.2)	3.6 (1.2)	3.8 (1.2)	4.0 (1.2)	4.0 (1.1)	< 0.001
HDL cholesterol, mmol/L	1.0 (0.3)	0.9 (0.3)	1.0 (0.3)	1.0 (0.3)	1.0 (0.3)	< 0.001
LDL cholesterol, mmol/L	2.2 (0.9)	2.0 (0.9)	2.1 (0.8)	2.3 (0.9)	2.3 (0.9)	< 0.001
AST, U/L	15.0 (11.0–20.0)	15.6 (11.4–21.8)	15.0 (11.1–20.7)	14.9 (11.0–19.5)	14.6 (10.9–19.3)	0.015
ALT, U/L	11.0 (7.2–17.5)	11.0 (7.0–18.0)	11.0 (7.6–17.0)	11 (7.3–17.5)	11.0 (7.3–18.0)	0.881
GGT, U/L	21 (14–35)	25.5 (16.1–45.0)	24.8 (15.7–39.8)	19.1 (14.0–31.0)	17.2 (12.7–28.0)	< 0.001
Bicarbonate, μmol/L	22.5 (4.3)	22.2 (4.3)	22.2 (4.2)	22.6 (4.4)	23.1 (4.1)	0.001
Corrected serum calcium, mmol/L	2.3 (0.2)	2.3 (0.2)	2.3 (0.2)	2.3 (0.2)	2.3 (0.2)	0.363
Serum phosphate, μmol/L	1.8 (0.6)	1.7 (0.6)	1.8 (0.6)	1.9 (0.7)	1.9 (0.6)	< 0.001
iPTH, pg/mL	241.4 (123.6–437.0)	218.3 (104.7–374.4)	240.0 (116.8–448.4)	252.8 (132.0–460.5)	259.6 (141.2–454.2)	0.001
CT data						
Log (TAC + 1)	4.4 (3.4)	6.5 (2.6)	5.1 (3.2)	3.7 (3.3)	2.5 (2.9)	< 0.001
Log (ATAC + 1)	0.5 (1.4)	0.8 (1.8)	0.6 (1.6)	0.3 (1.1)	0.2 (1.0)	< 0.001
Log (AoAC + 1)	3.8 (3.3)	5.8 (2.8)	4.5 (3.2)	3.1 (3.1)	1.9 (2.7)	< 0.001
Log (DTAC + 1)	3.0 (3.1)	4.8 (3.0)	3.6 (3.2)	2.4 (2.9)	1.4 (2.4)	< 0.001

*Note:* Data are presented as mean (SD), *n* (%) or median (IQR). Characteristics were compared using one‐way ANOVA or Kruskal–Wallis for continuous variables and *χ* ^2^ for categorical variables. *p* < 0.05 was considered statistically significant.

Abbreviations: ALT, alanine aminotransferase; AoAC, aortic arch calcification; AST, aspartate aminotransferase; ATAC, ascending thoracic aortic calcification; BMI, body mass index; BP, blood pressure; DTAC, descending thoracic aortic calcification; FPG, fasting plasma glucose; GGT, gamma‐glutamyl transferase; HDL, high‐density lipoprotein; iPTH, intact parathyroid hormone; LDL, low‐density lipoprotein; SMD, skeletal muscle radiodensity; TAC, thoracic aortic calcification; WBC, white blood cell count.

A total of 82.6% of participants (*n* = 2079) had TAC scores greater than zero; 21.8% (*n* = 548), 75.5% (*n* = 1901) and 68.9% (*n* = 1734) had ATAC, AoAC and DTAC scores greater than zero, respectively. The prevalence of significant TAC was 56.0% (*n* = 1410). Increasing quartiles of SMD and SMI both showed a significant decrease in the prevalence of significant TAC (*p* < 0.001) (Figure S2).

#### 3.1.2 Independent Associations of SMD and SMI With TAC

**FIGURE 1:**
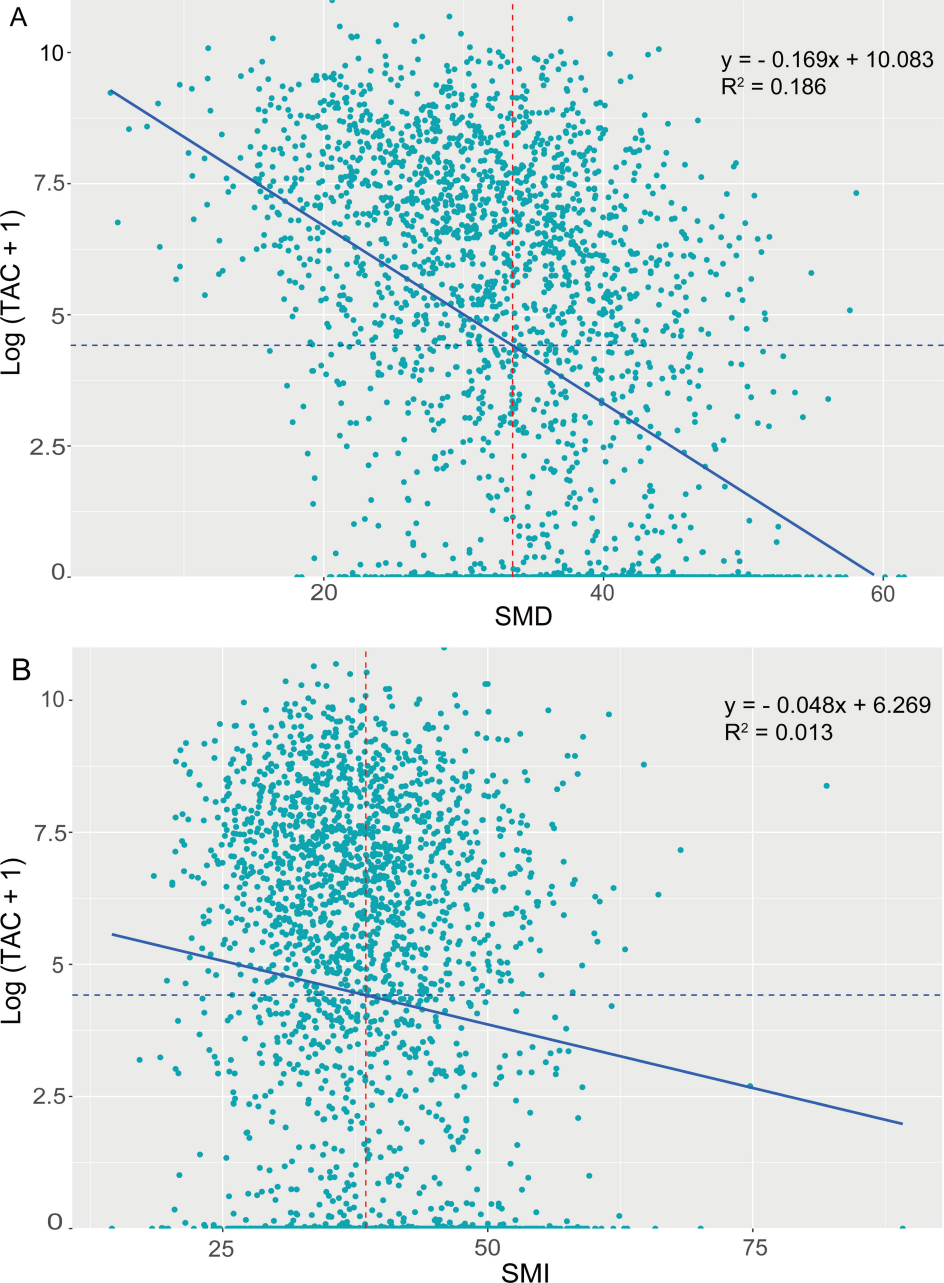
Correlation of SMD and SMI with TAC in dialysis patients. SMD and SMI exhibited significant inverse correlations with log (TAC + 1), as depicted in Graphs (A) and (B), respectively. The correlation with SMD was notably stronger, as indicated by a higher *R* ^2^ value. The blue dashed line represents the threshold for significant TAC, defined at a log (TAC + 1) value of 4.42, while the red dashed line indicates the corresponding SMI or SMD value at this threshold. TAC indicates thoracic aortic calcification; SMD, skeletal muscle radiodensity; SMI, skeletal muscle index.

**FIGURE 2:**
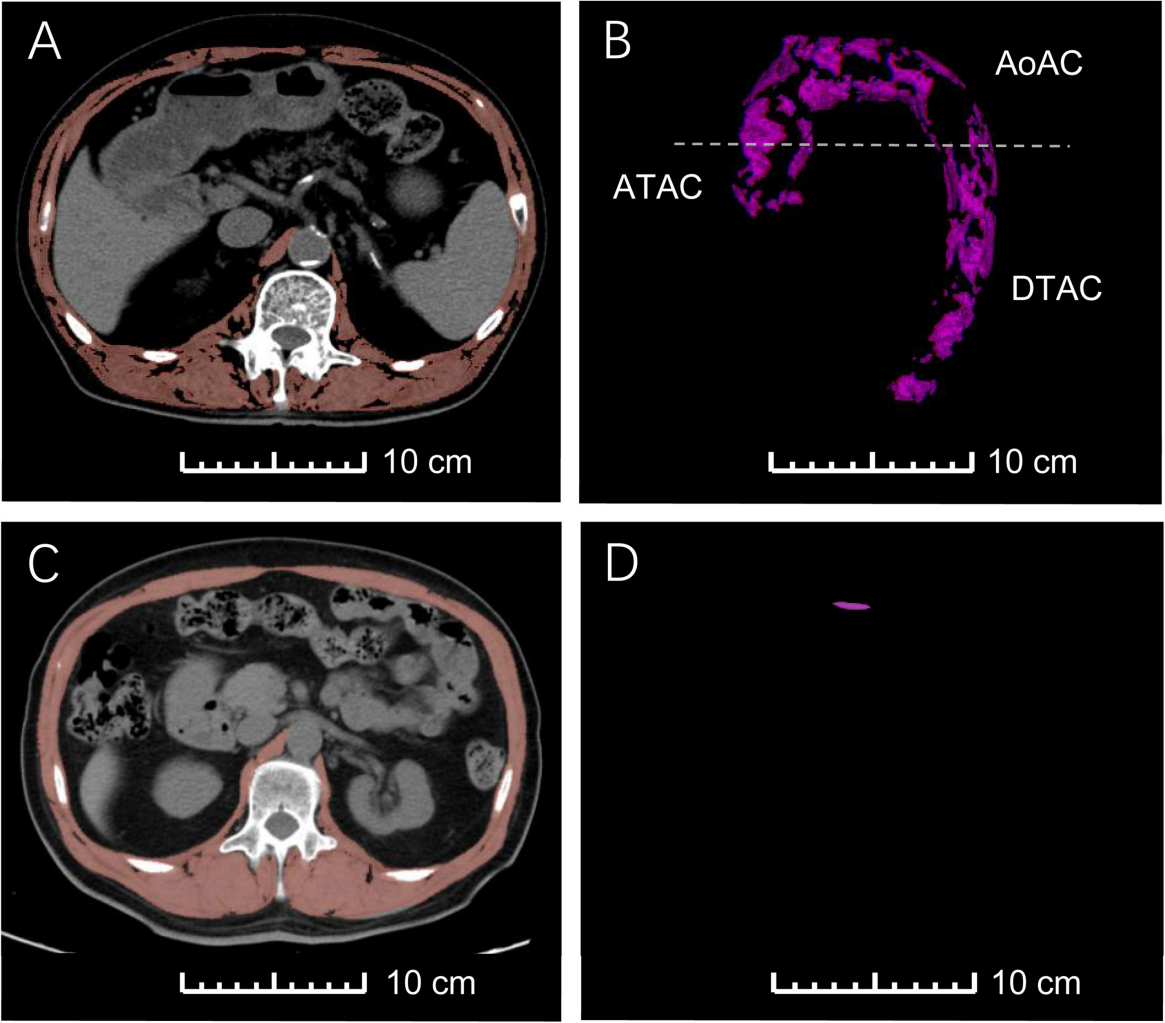
Illustrating the relationship between SMI, SMD and TAC with visual examples in CT imaging. An axial noncontrast composite CT image at the L1 level illustrates the segmentation of muscle (in red), and TAC can be evaluated by CT (in purple) within the same scan. Scale bars are shown in each panel, each measuring 10 cm, to provide a clear visual reference for the dimensions of the anatomical structures displayed in the CT images. (A) Body composition values in a 69‐year‐old man who underwent chest CT in 2022. Patient body composition values: L1 SMI = 29.3 cm^2^/m^2^, L1 SMD = 17.6 HU and (B) TAC = 14024.9 mm^3^. (C) Body composition values in another 69‐year‐old man who underwent chest CT in 2021, L1 SMI = 50.9 cm^2^/m^2^, L1 SMD = 39.0 HU and (D) TAC = 0.2 mm^3^. TAC indicates thoracic aortic calcification; ATAC, ascending thoracic aortic calcification; AoAC, aortic arch calcification; DTAC, descending thoracic aortic calcification; SMI, skeletal muscle index; SMD, skeletal muscle radiodensity.

In the unadjusted model, SMD as a continuous variable was significantly negatively correlated with TAC (Figure 1). After adjustment (Models 1–3), SMD quartile and SMD as a continuous value (per 1 SD increase in SMD) remained inversely associated with TAC, AoAC and DTAC but not with ATAC (Table 2 and Figure 2). Taking TAC as an example, in the fully adjusted model (Model 3), the regression coefficients (95% CI) of SMD for Q2, Q3 and Q4 compared with Q1 were −0.45 (−0.73 to −0.17), −0.91 (−1.21 to −0.61) and −1.08 (−1.42 to −0.75), respectively (*p* for trend < 0.001) (Table 2). An increase of 1 SD in SMD resulted in a decrease of 0.12 SD (95% CI, −0.16 to −0.08; *p* < 0.001) in TAC in Model 3.

**TABLE 2:** Regression coefficients (*β*) for association of SMD with TAC.

	SMD quartile separated by sex				*p* for trend	Continuous variable (per 1 SD increase in SMD)	*p*
	Q1 (lowest)	Q2	Q3	Q4 (highest)			
TAC							
Model 1	0 (ref.)	−0.43 (−0.74 to −0.13)	−0.99 (−1.31 to −0.67)	−1.36 (−1.71 to −1.01)	< 0.001	−0.16 (−0.20 to −0.12)	< 0.001
Model 2	0 (ref.)	−0.48 (−0.77 to −0.20)	−0.89 (−1.19 to −0.58)	−1.11 (−1.45 to −0.77)	< 0.001	−0.12 (−0.16 to −0.08)	< 0.001
Model 3	0 (ref.)	−0.45 (−0.73 to −0.17)	−0.91 (−1.21 to −0.61)	−1.08 (−1.42 to −0.75)	< 0.001	−0.12 (−0.16 to −0.08)	< 0.001
ATAC							
Model 1	0 (ref.)	−0.04 (−0.20 to 0.12)	−0.21 (−0.38 to −0.04)	−0.15 (−0.34 to 0.04)	0.044	−0.07 (−0.12 to −0.02)	0.004
Model 2	0 (ref.)	−0.04 (−0.20 to 0.12)	−0.18 (−0.35 to −0.01)	−0.09 (−0.28 to 0.10)	0.203	−0.05 (−0.10 to 0.00)	0.048
Model 3	0 (ref.)	−0.03 (−0.19 to 0.13)	−0.18 (−0.35 to −0.01)	−0.07 (−0.26 to 0.12)	0.250	−0.05 (−0.10 to 0.00)	0.065
AoAC							
Model 1	0 (ref.)	−0.45 (−0.75 to −0.15)	−1.01 (−1.33 to −0.69)	−1.43 (−1.78 to −1.08)	< 0.001	−0.17 (−0.21 to −0.13)	< 0.001
Model 2	0 (ref.)	−0.49 (−0.78 to −0.20)	−0.91 (−1.22 to −0.60)	−1.19 (−1.54 to −0.85)	< 0.001	−0.13 (−0.17 to −0.09)	< 0.001
Model 3	0 (ref.)	−0.46 (−0.75 to −0.18)	−0.93 (−1.24 to −0.62)	−1.17 (−1.52 to −0.83)	< 0.001	−0.13 (−0.17 to −0.09)	< 0.001
DTAC							
Model 1	0 (ref.)	−0.46 (−0.76 to −0.17)	−1.02 (−1.33 to −0.70)	−1.25 (−1.60 to −0.91)	< 0.001	−0.16 (−0.20 to −0.11)	< 0.001
Model 2	0 (ref.)	−0.55 (−0.84 to −0.26)	−1.00 (−1.30 to −0.69)	−1.13 (−1.47 to −0.79)	< 0.001	−0.13 (−0.17 to −0.09)	< 0.001
Model 3	0 (ref.)	−0.52 (−0.80 to −0.23)	−1.01 (−1.32 to −0.70)	−1.10 (−1.45 to −0.76)	< 0.001	−0.13 (−0.17 to −0.09)	< 0.001

*Note:* Model 1: Adjusted for SMI, age and sex. Model 2: Adjusted for all the covariates included in Model 1 and additionally adjusted for BMI, smoking history, dialysis duration, history of hypertension and history of diabetes. Model 3: Included all the covariates from Model 2 and additionally adjusted for log WBC, log (TG + 1), LDL cholesterol, log iPTH, serum phosphate, corrected serum calcium and vitamin D use.

Abbreviations: SMD indicates skeletal muscle radiodensity; SMI, skeletal muscle index; SD, standard deviation; BMI, body mass index; WBC, white blood cell count; TG, triglycerides; LDL, low‐density lipoprotein; iPTH, intact parathyroid hormone; TAC, thoracic aortic calcification; ATAC, ascending thoracic aortic calcification; AoAC, aortic arch calcification; DTAC, descending thoracic aortic calcification.

In the unadjusted model, SMI as a continuous variable was significantly negatively correlated with TAC (Figure 1). After adjustment (Models 1–3), SMI quartile and SMI as a continuous value (per 1 SD increase in SMI) remained inversely associated with TAC, AoAC and DTAC but not with ATAC (Table S2 and Figure 2).

### 3.2 Longitudinal Association Between SMD/SMI Separated by Sex and TAC Progression

#### 3.2.1 Participant Characteristics

The longitudinal cohort study comprised 544 individuals, with the mean (SD) age being 51.7 (13.3) years, males comprising 64.9% and the mean (SD) BMI at 23.6 (3.8) kg/m^2^. The mean (SD) follow‐up duration was 3.45 (1.82) years. At baseline, a total of 56.8% of patients initiating dialysis had TAC scores greater than zero; 6.6%, 52.4% and 43.8% had ATAC, AoAC and DTAC scores greater than zero, respectively. During the follow‐up, 85.7% of patients showed progression of TAC, with the median annualized absolute rate of progression being 96.98 mm^3^ per year. Using this median rate as a threshold, patients were categorized into rapid progressors (≥ 96.98 mm^3^; *n* = 272) and slow progressors (< 96.98 mm^3^; *n* = 272). Rapid progressors were typically older, had higher BMIs, showed more smoking history and a higher incidence of diabetes and hypertension (Table S3). They also exhibited elevated levels of WBC, FPG and GGT compared to those with slower progression. Additionally, it was observed that SMD was significantly lower in rapid progressors compared to slow progressors.

#### 3.2.2 Independent Associations of SMD and SMI With TAC Progression

**FIGURE 3:**
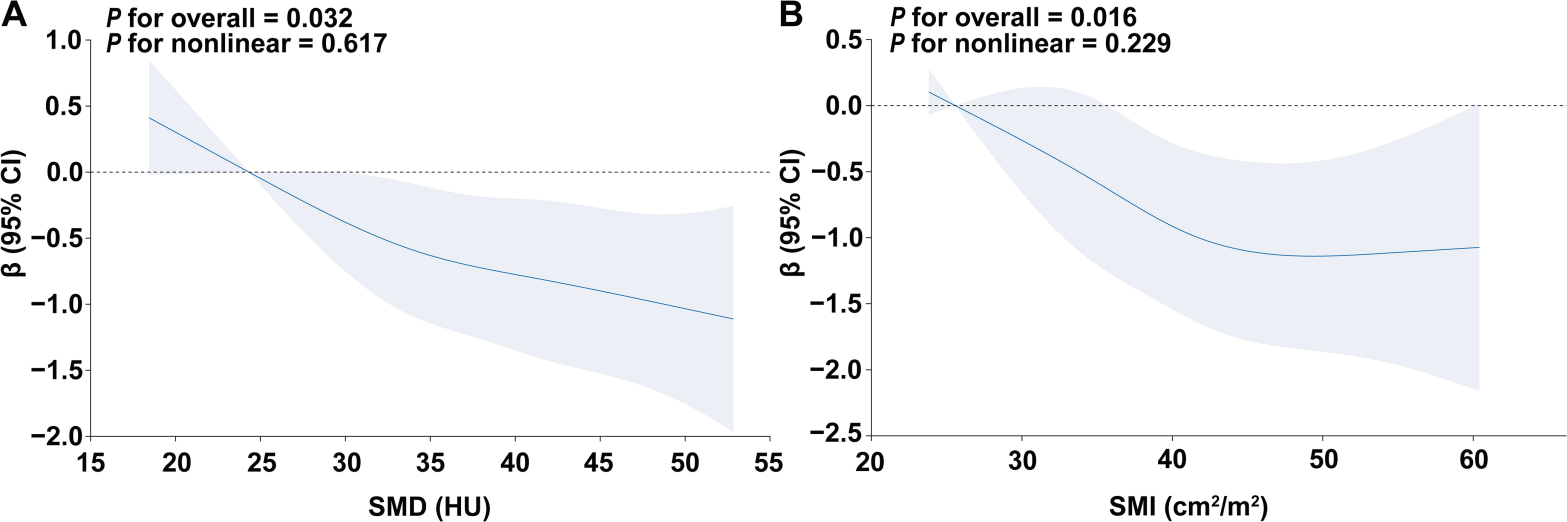
Restricted cubic splines analysis of SMD and SMI on TAC progression. Regression coefficients (*β*) of TAC progression with SMD (A) and SMI (B) depicted in fully adjusted models. These coefficients were derived after adjusting for age, sex, SMI (SMD model), SMD (SMI model), BMI, smoking history, history of hypertension, history of diabetes, log WBC, log (TG + 1), LDL cholesterol, log iPTH, serum phosphate, corrected serum calcium, vitamin D use and log (baseline TAC + 1). The shaded area represents the 95% confidence interval. SMD indicates skeletal muscle radiodensity; SMI, skeletal muscle index; TAC, thoracic aortic calcification; BMI, body mass index; WBC, white blood cell count; TG, triglycerides; LDL, low‐density lipoprotein; iPTH, intact parathyroid hormone.

After multivariable adjustment (Models 1–3), SMD quartile and SMD as a continuous value (per 1 SD increase in SMD) showed significantly inverse association with TAC progression, AoAC progression and DTAC progression but not with ATAC progression (Table 3). Taking TAC progression as an example, in Model 3, compared with the reference group (the first quartile), the *β* of SMD was −0.52 (95% CI, −1.00 to −0.04; *p* = 0.034; *E* value, 1.65 [upper CI, 1.00]) in the second quartile, −0.52 (95% CI, −1.03 to −0.02; *p* = 0.043; *E* value, 1.65 [upper CI, 1.00]) in the third quartile and −0.83 (95% CI, −1.37 to −0.29; *p* = 0.003; *E* value, 1.96 [upper CI, 1.43]) in the fourth quartile (Table 3). An increase of 1 SD in SMD resulted in a decrease of 0.10 SD (95% CI, −0.17 to −0.02; *p* = 0.011) in TAC progression in Model 3. Restricted cubic splines demonstrated that as SMD increased, the TAC progression decreased, showing no evidence of non‐linearity (*p* for non‐linear = 0.617) (Figure 3A).

**TABLE 3:** Regression coefficients (*β*) for association of SMD with TAC progression.

	SMD quartile separated by sex				*p* for trend	Continuous variable (per 1 SD increase in SMD)	*p*
	Q1 (lowest)	Q2	Q3	Q4 (highest)			
TAC							
Model 1	0 (ref.)	−0.72 (−1.27 to −0.18)	−1.00 (−1.56 to −0.44)	−1.11 (−1.72 to −0.50)	< 0.001	−0.14 (−0.22 to −0.06)	0.001
Model 2	0 (ref.)	−0.68 (−1.22 to −0.14)	−0.86 (−1.43 to −0.29)	−1.07 (−1.68 to −0.46)	0.001	−0.13 (−0.21 to −0.05)	0.002
Model 3	0 (ref.)	−0.52 (−1.00 to −0.04)	−0.52 (−1.03 to −0.02)	−0.83 (−1.37 to −0.29)	0.005	−0.10 (−0.17 to −0.02)	0.011
ATAC							
Model 1	0 (ref.)	−0.12 (−0.43 to 0.19)	−0.10 (−0.42 to 0.22)	−0.13 (−0.47 to 0.21)	0.503	−0.01 (−0.11 to 0.09)	0.857
Model 2	0 (ref.)	−0.10 (−0.41 to 0.20)	−0.05 (−0.38 to 0.27)	−0.12 (−0.47 to 0.23)	0.589	0.00 (−0.10 to 0.10)	0.990
Model 3	0 (ref.)	−0.24 (−0.53 to 0.05)	−0.12 (−0.42 to 0.18)	−0.15 (−0.47 to 0.18)	0.542	0.00 (−0.09 to 0.10)	0.946
AoAC							
Model 1	0 (ref.)	−0.68 (−1.21 to −0.15)	−1.06 (−1.61 to −0.51)	−1.03 (−1.63 to −0.44)	< 0.001	−0.14 (−0.22 to −0.06)	0.001
Model 2	0 (ref.)	−0.67 (−1.20 to −0.13)	−0.99 (−1.55 to −0.43)	−1.04 (−1.64 to −0.44)	< 0.001	−0.14 (−0.22 to −0.05)	0.002
Model 3	0 (ref.)	−0.58 (−1.05 to −0.10)	−0.76 (−1.26 to −0.26)	−0.90 (−1.44 to −0.37)	0.001	−0.11 (−0.18 to −0.03)	0.006
DTAC							
Model 1	0 (ref.)	−0.70 (−1.24 to −0.16)	−1.06 (−1.61 to −0.50)	−1.40 (−2.00 to −0.79)	< 0.001	−0.19 (−0.27 to −0.10)	< 0.001
Model 2	0 (ref.)	−0.68 (−1.22 to −0.14)	−0.98 (−1.55 to −0.41)	−1.38 (−1.99 to −0.77)	< 0.001	−0.18 (−0.27 to −0.09)	< 0.001
Model 3	0 (ref.)	−0.50 (−0.98 to −0.02)	−0.71 (−1.21 to −0.20)	−1.15 (−1.69 to −0.60)	< 0.001	−0.15 (−0.23 to −0.08)	< 0.001

*Note:* Model 1: Adjusted for SMI, age and sex. Model 2: Adjusted for all the covariates included in Model 1 and additionally adjusted for BMI, smoking history, history of hypertension and history of diabetes. Model 3: Included all the covariates from Model 2 and additionally adjusted for log WBC, log (TG + 1), LDL cholesterol, log iPTH, serum phosphate, corrected serum calcium, vitamin D use and log (baseline calcification score + 1).

Abbreviations: AoAC, aortic arch calcification; ATAC, ascending thoracic aortic calcification; BMI, body mass index; DTAC, descending thoracic aortic calcification; iPTH, intact parathyroid hormone; LDL, low‐density lipoprotein; SD, standard deviation; SMD, skeletal muscle radiodensity; SMI, skeletal muscle index; TAC, thoracic aortic calcification; TG, triglycerides; WBC, white blood cell count.

After multivariable adjustment (Models 1–3), SMI quartile and SMI as a continuous value (per 1 SD increase in SMI) showed significantly inverse association with TAC progression and DTAC progression but not with ATAC progression or AoAC progression (Table S4). Restricted cubic splines demonstrated that as SMI increased, the TAC progression decreased, showing no evidence of non‐linearity (*p* for non‐linear = 0.229) (Figure 3B).

The adjusted *R* ^2^ and AIC values for Models 1–3 are presented in Table 4. Overall, the adjusted *R* ^2^ for the TAC progression model increased from Model 1 to Model 3, while the AIC decreased, indicating improved model fit. Specifically, Model 3, which incorporated all covariates, demonstrated an adjusted *R* ^2^ of 0.5326 (vs. 0.3723 in Model 1 and 0.3821 in Model 2) and an AIC of 2263.14 (vs. 2411.75 in Model 1 and 2407.15 in Model 2). Consistent patterns were observed in the regression models for AoAC, ATAC and DTAC progression, with Model 3 showing significantly higher adjusted *R* ^2^ values and lower AIC compared to Models 1 and 2.

**TABLE 4:** Model fit assessment for the association of SMD/SMI with TAC progression.

Dependent variable	Model	Adjusted *R* ^2^	AIC
TAC	Model 1	0.3723	2411.75
	Model 2	0.3821	2407.15
	Model 3	0.5326	2263.14
ATAC	Model 1	0.0647	1787.15
	Model 2	0.0658	1790.49
	Model 3	0.2141	1704.22
AoAC	Model 1	0.3501	2387.03
	Model 2	0.3546	2387.17
	Model 3	0.5039	2251.87
DTAC	Model 1	0.3300	2399.36
	Model 2	0.3309	2402.55
	Model 3	0.4887	2264.10

*Note:* Model 1 was adjusted for SMI (SMD model), SMD (SMI model), age and sex. Model 2: Adjusted for all the covariates included in Model 1 and additionally adjusted for BMI, smoking history, history of hypertension and history of diabetes. Model 3: Included all the covariates from Model 2 and additionally adjusted for log WBC, log (TG + 1), LDL cholesterol, log iPTH, serum phosphate, corrected serum calcium, vitamin D use and log (baseline calcification score + 1).

Abbreviations: AIC, Akaike information criterion; AoAC, aortic arch calcification; ATAC, ascending thoracic aortic calcification; BMI, body mass index; DTAC, descending thoracic aortic calcification; iPTH, intact parathyroid hormone; LDL, low‐density lipoprotein; SMD, skeletal muscle radiodensity; SMI, skeletal muscle index; TAC, thoracic aortic calcification; TG, triglycerides; WBC, white blood cell count.

**FIGURE 4:**
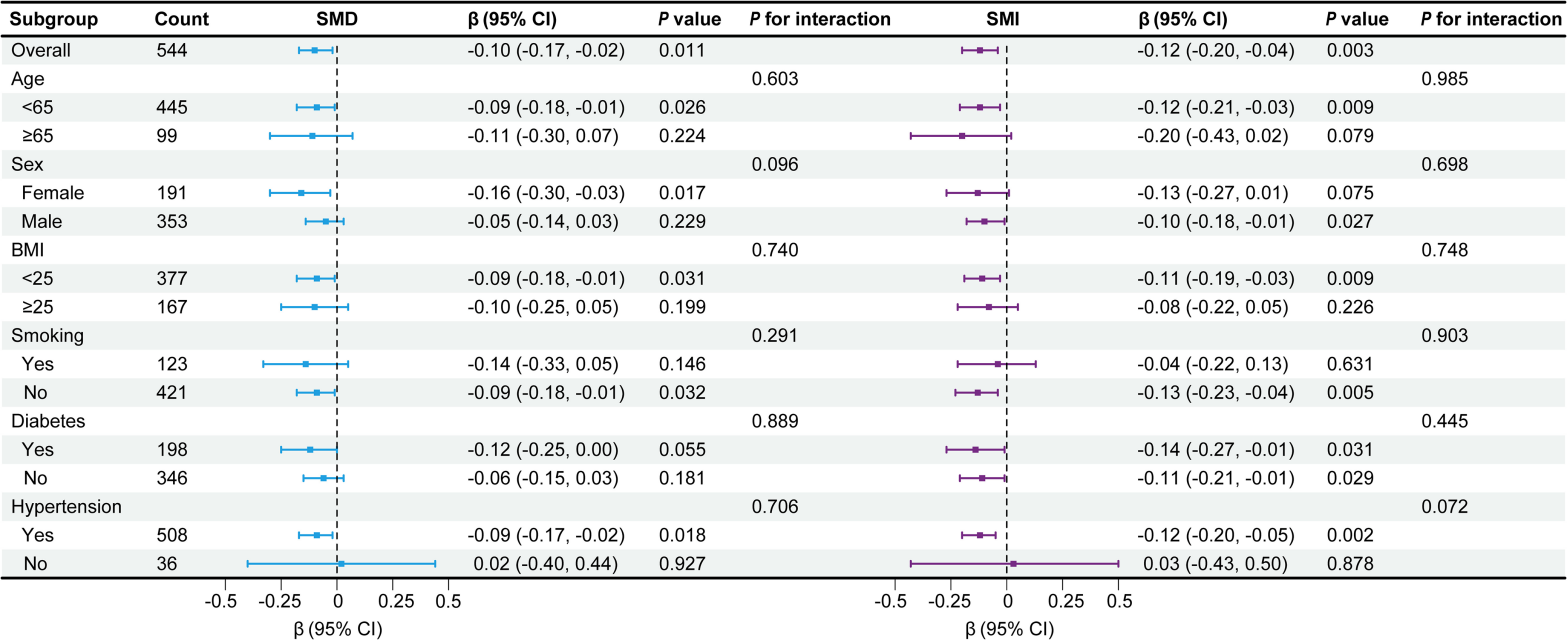
Subgroup analyses of the association between SMD/SMI and TAC. Estimated from multivariable linear regression. The multivariable model was adjusted for age, sex, SMI (SMD model), SMD (SMI model), BMI, smoking history, history of hypertension, history of diabetes, log WBC, log (TG + 1), LDL cholesterol, log iPTH, serum phosphate, corrected serum calcium, vitamin D use and log (baseline TAC + 1). All *β* are standardized coefficients. SMD indicates skeletal muscle radiodensity; SMI, skeletal muscle index; TAC, thoracic aortic calcification; CI, confidence interval; BMI, body mass index; WBC, white blood cell count; TG, triglycerides; LDL, low‐density lipoprotein; iPTH, intact parathyroid hormone.

In the subgroup analysis, the standardized coefficients for SMD/SMI were mostly statistically significant (Figure 4). After adjusting for multiple testing, no significant interactions were found between SMI, SMD or any other strata variables with TAC progression (all *p* for interaction > 0.05).

#### 3.2.3 Sensitivity Analysis

First, after excluding patients with stroke and CHD, the relationship of SMD and SMI with TAC progression was further evaluated and found to be generally stable (Tables S5–S8). Second, patients were divided into rapid and slow TAC progressors based on the median annualized rate. After multivariable adjustment, both higher SMD and SMI were associated with lower odds of rapid progression in TAC (Figure S3). Last, to assess the impact of unobservable variables on the effect estimates, *E* values were calculated. All *E* values were greater than 1, which suggested that considerable unmeasured confounding would be needed to explain away the effect estimate. Details on *E* values are provided in Method S3.

## 4 Discussion

This is the first study to utilize a large multicentre sample to assess the relationships of skeletal muscle mass and quality with TAC and its progression in dialysis patients. In a cross‐sectional analysis, we found a negative association of SMD and SMI with TAC. Furthermore, higher SMD and higher SMI were independently associated with lower TAC progression in initial‐dialysis patients in a longitudinal analysis. These findings suggest that assessing muscle mass and quality could be crucial for predicting and managing vascular calcification and its progression in dialysis patients.

Prior research has demonstrated a correlation between sarcopenia, as determined by the psoas muscle index measured via CT images, and all‐cause mortality in dialysis patients [25, 26, 27]. Our recent study revealed that low SMD (at the L1 level) assessed by CT, reflecting a decline in skeletal muscle quality, was a risk factor for all‐cause mortality and CV events in initial‐dialysis patients over a 3‐year follow‐up period [16]. Nevertheless, the mechanisms underlying the heightened CV risk in dialysis patients with low SMI and SMD remain unclear. It is commonly acknowledged that TAC is a prevalent CV complication in individuals receiving dialysis, with TAC identified in 82.6% of patients in this study. TAC not only serves as a marker for systemic atherosclerosis but also contributes to the progression of arteriosclerosis, ultimately leading to end‐organ damage [28, 29]. Our findings indicated that SMD and SMI had significant inverse associations with TAC and its progression in dialysis patients. Although this study has not fully determined all possible mechanisms associated with TAC, we surmise that skeletal muscle quality and skeletal muscle mass might be implicated in TAC progression through another mechanism, independent of traditional CV risk factors, suggesting the distinct role of skeletal muscle quality and mass in promoting TAC. Therefore, we speculate that low skeletal muscle quality and low skeletal muscle mass may influence the risk of CV disease by affecting the calcification process of the thoracic aorta.

There is a close relationship between skeletal muscle and vascular calcification. Skeletal muscle is not only a crucial organ for glucose uptake and metabolism but also secretes and expresses various myokines. Reduced skeletal muscle leads to insulin resistance and endothelial dysfunction, increasing vascular calcification risk [30]. Additionally, this reduction may lead to decreased irisin, which potentially inhibits vascular calcification by preventing osteogenic transformation in vascular smooth muscle cells, enhancing mitochondrial function, promoting autophagy and suppressing cell apoptosis [31, 32]. In addition, alterations in skeletal muscle function may impact the secretion of muscle‐derived exosomes, which could lead to improvements in insulin sensitivity, reduction in visceral fat deposition and inhibition of atherosclerosis progression, potentially contributing to the mitigation of vascular calcification [33, 34]. We found that patients with lower SMD had higher TAC and FPG levels, suggesting that TAC in dialysis patients with lower skeletal muscle quality may be associated with insulin resistance.

The current study found higher SMD correlated with better health markers, while higher SMI indicated greater disease risk, higher BMI and lower ALB levels. Following multivariate adjustment in the cross‐sectional study, SMD showed a significant negative correlation with TAC in dialysis patients, with this inverse relationship being more pronounced than that observed with SMI. In the longitudinal study, it was consistently observed that higher SMD was associated with lower rates of TAC progression across all models. Conversely, the association between SMI and TAC progression was less stable, particularly in the second and fourth quartiles. Some studies have suggested that muscle quality might be more important than its mass in predicting adverse outcomes in dialysis patients [35, 36]. However, the reason for this phenomenon is not clear. Some believe that an increase in muscle area may be related to more fat infiltration within the muscle [37], but more work is needed to explore the specific mechanisms. Interventions such as resistance/strength training could improve both of these muscle characteristics [38].

The distribution of calcification within the aorta frequently displayed notable heterogeneity [39]. Various studies have demonstrated that calcification in different aortic segments had varying predictive values for the incidence and mortality of CV and non‐CV diseases [7, 40, 41], and there might be differences in related CV risk factors [42]. Our investigation thus evaluated TAC, ATAC, AoAC and DTAC both comprehensively and individually. Cross‐sectional results showed SMD and SMI correlation with AoAC and DTAC aligned with TAC, excluding ATAC. Longitudinal analysis revealed distinct patterns in the correlation of SMD and SMI with the progression of various segments of TAC. Both SMD and SMI were significantly negatively correlated with overall TAC progression and DTAC progression, underscoring their role in mitigating calcification across comprehensive and specific lower segments of the thoracic aorta. However, SMD alone showed a significant negative correlation with AoAC progression, suggesting its unique protective role in this specific aortic segment, unlike SMI, which did not demonstrate such an association. Neither SMD nor SMI showed significant associations with ATAC progression, possibly due to its lower prevalence rate. In the current study, only 6.6% of participants were found to have ATAC, a low percentage similar to other studies [43], which may have limited our ability to detect certain associations. Therefore, our study highlighted a differential sensitivity of aortic segments to the protective effects of muscle quality and mass, providing key insights for developing more targeted CV disease prevention and treatment strategies in dialysis patients.

This study has several limitations. First, despite employing a multivariate model that adjusted for a wider range of potential confounders, our research still harbours bias due to potential unmeasured confounders, such as the lack of information on physical function and physical activity levels. To mitigate omitted variable bias, we employed the *E* value to assess the impact of unobservable variables on the estimated effect. Second, although we evaluated the volume of TAC, we did not examine other key characteristics of calcification, such as density and area. Third, only Chinese dialysis patients were included, limiting generalizability to Western populations. Fourth, because chest CT was not performed in all patients, there is a potential selection bias. However, this study rigorously controlled for potential confounders and adjusted covariates meticulously. The large sample size, precise CT measurements and automated software use ensure high repeatability. Thus, the SMD and SMI relationships with TAC progression may reflect broader trends in dialysis patients. Lastly, although our observational study has to some extent demonstrated that higher SMD and higher SMI were associated with lower TAC progression in initial‐dialysis patients, establishing a more definitive causal relationship needs further exploration through controlled intervention studies.

## 5 Conclusions

In summary, this study demonstrated that SMD and SMI had significant negative associations with TAC in dialysis patients. It also highlighted that higher SMD and higher SMI were independently associated with lower TAC progression in initial‐dialysis patients. Considering the established link between TAC and systemic arteriosclerosis, as well as CV incidents, it suggests that muscle quality and mass might influence the development of CV diseases in dialysis patients through affecting the progression of TAC. This implies that improving body composition could potentially enhance patient outcomes by lowering the incidence of CV events.

## Ethics Statement

Ethical approval for this research was granted by Zhongda Hospital's medical research ethics committee (approval number: 2023ZDSYLL172‐P01), with registration in the Chinese Clinical Trial Registry (registration number: ChiCTR2300075231), and was conducted according to the Declaration of Helsinki. The requirement for signed informed consent was waived. The authors of this manuscript certify that they comply with the ethical guidelines for authorship and publishing in the *Journal of Cachexia, Sarcopenia and Muscle* [44].

## Conflicts of Interest

The authors declare no conflicts of interest.

## Supporting information


**Figure S1.** Flowchart of patient selection in the cross‐sectional study.
**Figure S2.** Comparison of significant TAC prevalence across quartiles of SMI and SMD separated by sex.
**Figure S3.** Odds ratio (95% confidence intervals) of rapid TAC progression according to SMD/SMI.
**Table S1.** Baseline characteristics of total subjects according to the SMI quartile separated by sex.
**Table S2.** Regression coefficients (*β*) for association of SMI with TAC.
**Table S3.** Baseline characteristics of subjects by TAC progression.
**Table S4.** Regression coefficients (*β*) for association of SMI with TAC progression.
**Table S5.** Regression coefficients (*β*) for association of SMD with TAC progression after excluding patients diagnosed with stroke.
**Table S6.** Regression coefficients (*β*) for association of SMI with TAC progression after excluding patients diagnosed with stroke.
**Table S7.** Regression coefficients (*β*) for association of SMD with TAC progression after excluding patients diagnosed with stroke or coronary heart disease.
**Table S8.** Regression coefficients (*β*) for association of SMI with TAC progression after excluding patients diagnosed with stroke or coronary heart disease.

## Data Availability

Access to anonymized data may be requested from the corresponding author within reasonable limits.
