# Supplementary material for: Association of Muscle Radiodensity and Muscle Mass With Thoracic Aortic Calcification Progression in Dialysis Patients
Source: J Cachexia Sarcopenia Muscle. 2025 Apr 17;16(2):e13813. doi: 10.1002/jcsm.13813 (PMC12003956; doi:10.1002/jcsm.13813)
Supplement: Supplementary file 1 — Figure S1. Flowchart of patient selection in the cross‐sectional study. Figure S2. Comparison of significant TAC prevalence across quartiles of SMI and SMD separated by sex. Figure S3. Odds ratio (95% confidence intervals) of rapid TAC progression according to SMD/SMI. Table S1. Baseline characteristics of total subjects according to the SMI quartile separated by sex. Table S2. Regression coefficients (β) for association of SMI with TAC. Table S3. Baseline characteristics of subjects by TAC progression. Table S4. Regression coefficients (β) for association of SMI with TAC progression. Table S5. Regression coefficients (β) for association of SMD with TAC progression after excluding patients diagnosed with stroke. Table S6. Regression coefficients (β) for association of SMI with TAC progression after excluding patients diagnosed with stroke. Table S7. Regression coefficients (β) for association of SMD with TAC progression after excluding patients diagnosed with stroke or coronary heart disease. Table S8. Regression coefficients (β) for association of SMI with TAC progression after excluding patients diagnosed with stroke or coronary heart disease. [file JCSM-16-e13813-s001.docx]

**Method S1.** Assessment of Thoracic Aortic Calcification

**Method S2.** Definitions of Diabetes, Hypertension, Coronary Heart Disease, Hyperlipidemia, and Stroke

**Method S3.** Sensitivity Analysis for Unmeasured Confounding

**Fig.S1** Flowchart of Patient Selection in the Cross-sectional Study

**Fig.S2** Comparison of Significant TAC Prevalence across Quartiles of SMI and SMD Separated by Sex

**Fig.S3** Odds Ratio (95% confidence intervals) of Rapid TAC Progression according to SMD/SMI

**Table S1.** Baseline Characteristics of Total Subjects According to the SMI Quartile Separated by Sex

**Table S2.** Regression Coefficients (β) for Association of SMI with TAC

**Table S3.** Baseline Characteristics of Subjects by TAC Progression

**Table S4.** Regression Coefficients (β) for Association of SMI with TAC Progression

**Table S5.** Regression Coefficients (β) for Association of SMD with TAC Progression after Excluding Patients Diagnosed with Stroke

**Table S6.** Regression Coefficients (β) for Association of SMI with TAC Progression after Excluding Patients Diagnosed with Stroke

**Table S7.** Regression Coefficients (β) for Association of SMD with TAC Progression after Excluding Patients Diagnosed with Stroke or Coronary Heart Disease

**Table S8.** Regression Coefficients (β) for Association of SMI with TAC Progression after Excluding Patients Diagnosed with Stroke or Coronary Heart Disease

**Method S1.** Assessment of Thoracic Aortic Calcification

The thoracic aorta refers to the portion of the aorta that runs along the thoracic cavity, extending from the aortic root to the level of the cardiac apex. The thoracic aorta is divided into three segments: the ascending thoracic aorta (ATA), the aortic arch (AoA), and the descending thoracic aorta (DTA). The specific segmentation is as follows:

(1) ATA: This segment extends from the aortic root to the level of the lower edge of pulmonary artery bifurcation.

(2) AoA: The aortic arch segment is situated between the terminal end of the ATA and the beginning of the DTA.

(3) DTA: This segment extends from the lower edge of pulmonary artery bifurcation to the level of the cardiac apex (Current Atherosclerosis Reports. 2019; 21(12):51).

**Method S2.** Definitions of Diabetes, Hypertension, Coronary Heart Disease, Hyperlipidemia, and Stroke

Diabetes was identified by a fasting plasma glucose level at or above 126mg/dL (to convert glucose to mmol/L, multiply by 0.0555), a nonfasting plasma glucose level at or above 200 mg/dL, or through self-reported diabetes medication use. Blood pressure was measured in the supine position after a rest of 15-20 minutes, taken from the arm without arteriovenous fistula or shunt. Hypertension was defined as a systolic pressure greater than 140 mm Hg, a diastolic pressure greater than 90 mm Hg, or current use of antihypertensive medications. Coronary heart disease was defined as any condition including angina, myocardial infarction, coronary artery stenosis, percutaneous coronary intervention, or coronary artery bypass surgery. Dyslipidemia was characterized by having total cholesterol levels of 5.2 mmol/L (200 mg/dL) or higher, and LDL-cholesterol levels of 3.5 mmol/L (135 mg/dL) or higher, or by being uncontrolled despite treatment with the highest tolerated doses of other lipid-lowering medications for at least four weeks. Stroke was identified by the sudden appearance of a neurological deficit, headache, or other nonvascular symptoms, confirmed by a clinically significant lesion on brain imaging lasting more than 24 hours, or by death occurring within 24 hours.

**Method S3.** Sensitivity Analysis for Unmeasured Confounding

Additional sensitivity analysis was conducted using the E-value methodology of VanderWeele and Ding (Annals of Internal Medicine, 2017 Aug 15;167(4):268−74). This method calculates the minimum strength of association an unmeasured confounder would need with both SMD/SMI and the annualized absolute rate of TAC progression to overcome the statistically significant effect observed in our study where residual confounding is a potential problem. This calculation is derived from the regression coefficients obtained from our fully adjusted models.

For the current study, higher SMD and SMI quartiles were significantly associated with lower rates of annualized absolute TAC progression, as indicated by negative β coefficients. Specifically, compared with the reference group (the first quartile), the β of SMD in the second quartile was −0.52 (95% CI, −1.00–−0.04; *P* = 0.034), in the third quartile was −0.52 (95% CI, −1.03–−0.02; *P*=0.043), and in the fourth quartile was −0.83 (95% CI, −1.37–−0.29; *P* = 0.003) **(Table 3 of the main article)**. The E-values for the point estimates were 1.65 for both the second and third quartiles, and 1.96 for the fourth quartile, with upper confidence interval limits of 1.00 for the second and third quartiles, and 1.43 for the fourth quartile, respectively. Therefore, taking the second quartile as an example, following the methodology outlined by VanderWeele and Ding, the observed standardized regression coefficient of −0.52 could be explained by an unmeasured confounder associated with SMD increases and reduced TAC progression by a ratio of approximately 1.65. Moreover, above-mentioned E-values suggest that only stronger unmeasured confounding could explain these associations**,** yet it is unlikely that such substantial confounding exists in our study. Similarly, compared with the reference group (the first quartile), the β of SMI was −0.71 (95% CI, −1.20–−0.23; *P* = 0.004; E-value, 1.84 [upper CI, 1.31]) in the third quartile, and −0.58 (95% CI, −1.13–−0.03; *P* = 0.038; E-value, 1.71 [upper CI, 1.13]) in the fourth quartile **(Supplementary Table S4)**.


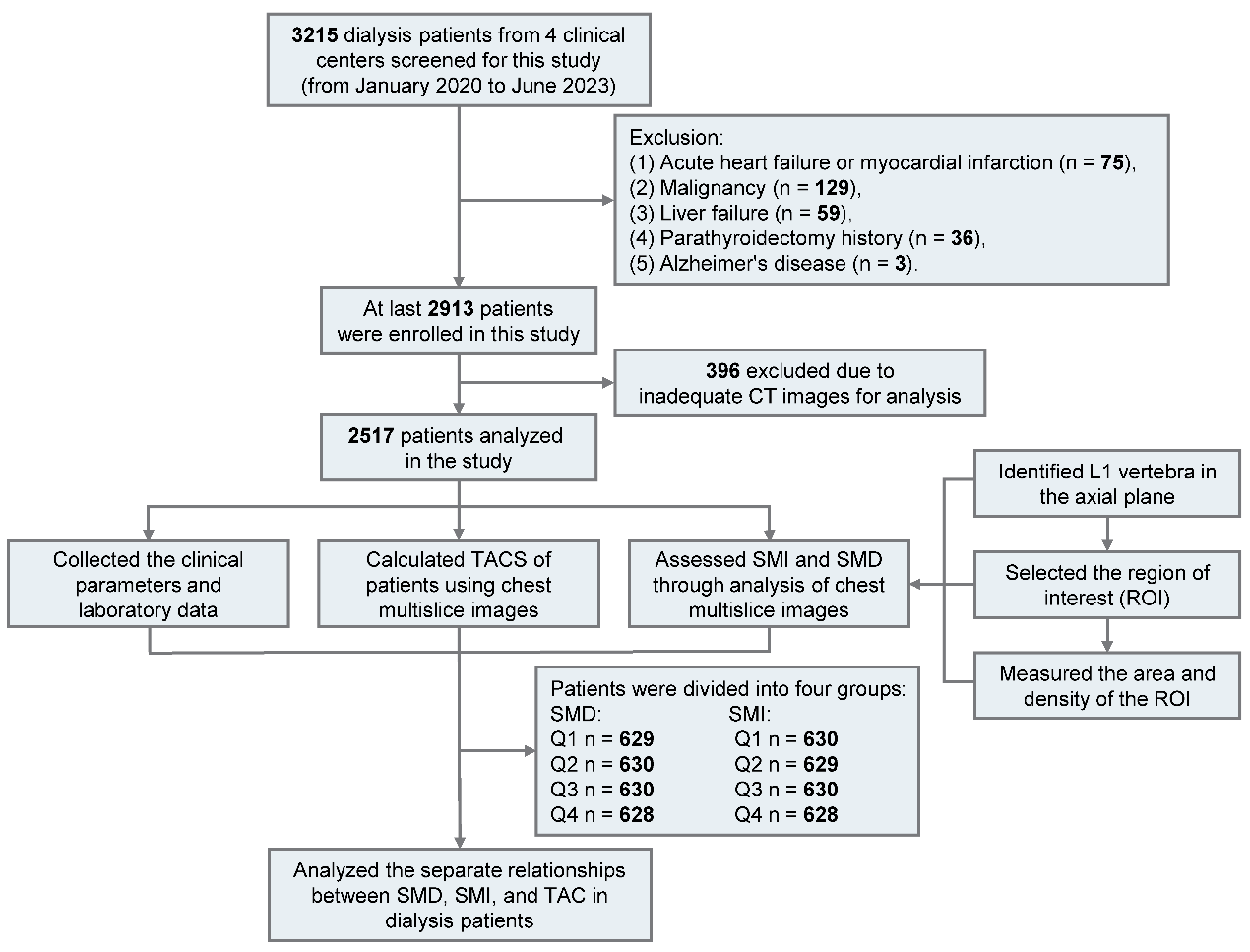


**Fig.S1 Flowchart of Patient Selection in the Cross-sectional Study.**

From 3215 dialysis patients, 2517 were eligible for inclusion. TAC indicates thoracic aortic calcification; SMD, skeletal muscle radiodensity; and SMI, skeletal muscle index.


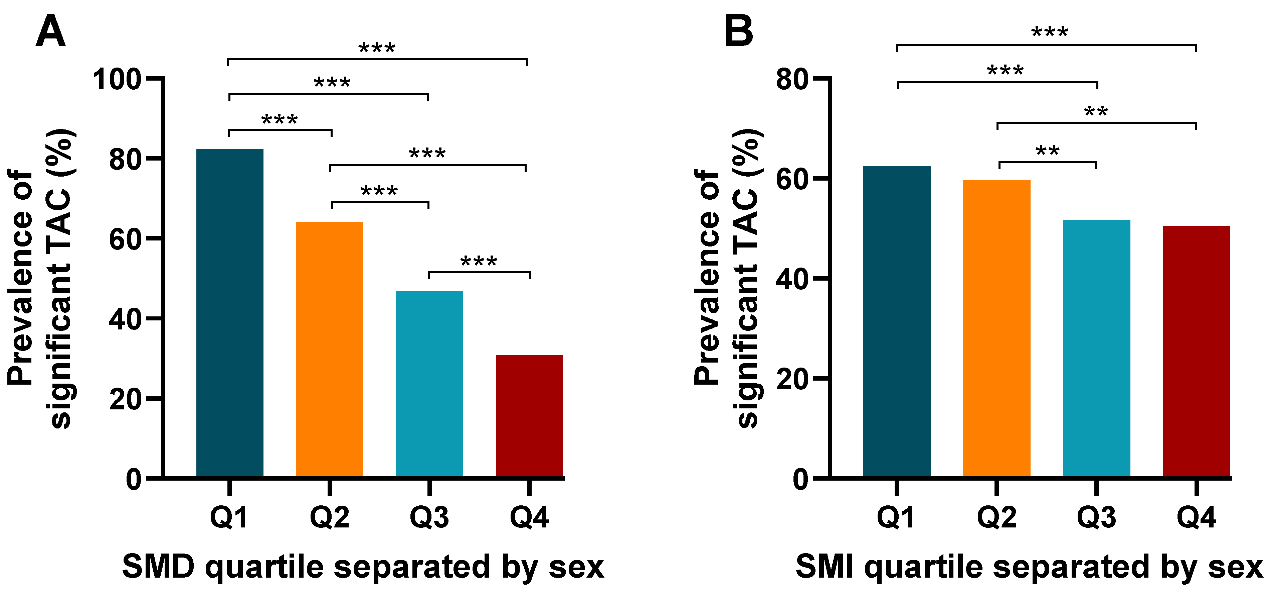


**Fig.S2 Comparison of Significant TAC Prevalence across Quartiles of SMI and SMD Separated by Sex.**

Figure S2 illustrates the comparison between the quartiles of SMI and SMD and the prevalence of significant TAC. The data revealed a higher prevalence of significant TAC in the lower quartiles of SMD. As the quartiles of SMD increased, there was a significant decrease in the prevalence of significant TAC (*P*<0.001). Similarly, with the increase in the quartiles of SMI, the prevalence of significant TAC decreased (*P*<0.001), although not all differences between the quartiles of SMI reached statistical significance. Statistical analysis was conducted using ANOVA with post-hoc pairwise comparisons to assess differences between quartiles. Significance levels were indicated as follows: **P*<0.05, ***P*<0.01, ****P*<0.001. TAC indicates thoracic aortic calcification; SMI, skeletal muscle index; and SMD, skeletal muscle radiodensity.


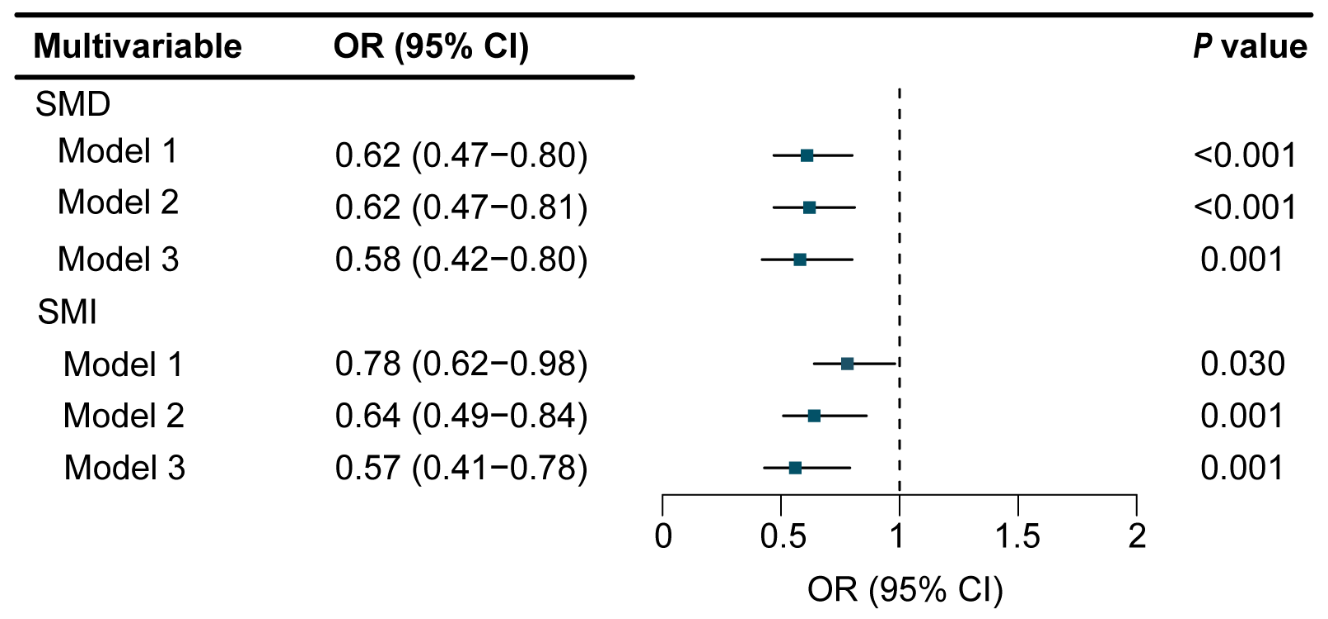


**Fig.S3 Odds Ratio (95% confidence intervals) of Rapid TAC Progression according to SMD/SMI.**

Model 1: adjusted for SMI (SMD models), SMD (SMI models), age, and sex. Model 2: adjusted for all the covariates included in model 1 and additionally adjusted for BMI, smoking history, history of hypertension, history of diabetes. Model 3: included all the covariates from model 2 and additionally adjusted for log WBC, log (TG + 1), LDL cholesterol, log iPTH, serum phosphate, corrected serum calcium, Vitamin D use, and log (baseline TAC + 1). SMI indicates skeletal muscle index; SMD, skeletal muscle radiodensity; BMI, body mass index; WBC, white blood cell count; TG, triglycerides; LDL, low−density lipoprotein; iPTH, intact parathyroid hormone; TAC, thoracic aortic calcification. The SMD/SMI variation is expressed per 1 standard deviation increase.

**Table S1.** **Baseline Characteristics of Total Subjects According to the SMI Quartile Separated by Sex**

| Characteristic |  | SMI quartile separated by sex | | | |  |
| --- | --- | --- | --- | --- | --- | --- |
|  | Over all | Q1 | Q2 | Q3 | Q4 | *P* value |
| Number | 2517 | 630 | 629 | 630 | 628 |  |
| SMI, cm^2^/m^2^ |  |  |  |  |  |  |
| Men | 41.2 (8.0) | 31.9 (3.7) | 38.3 (1.3) | 43.2 (1.6) | 51.6 (5.2) |  |
| Women | 34.3 (6.6) | 26.6 (2.6) | 31.8 (1.1) | 35.8 (1.3) | 43.2 (4.6) |  |
| Age, years | 54.8 (14.0) | 56.8 (14.4) | 56.1 (13.9) | 54.0 (13.6) | 52.2 (13.6) | <0.001 |
| Systolic BP, mm Hg | 144.7 (25.4) | 140.0 (25.9) | 144.4 (25.5) | 145.2 (24.4) | 149.4 (25.1) | <0.001 |
| Diastolic BP, mm Hg | 85.2 (15.5) | 83.3 (15.3) | 85.6 (15.4) | 85.4 (15.6) | 86.5 (15.8) | 0.002 |
| BMI, kg/m^2^ | 23.0 (3.8) | 20.4 (3.0) | 22.2 (3.1) | 23.6 (3.0) | 25.9 (3.9) | <0.001 |
| Smoking history, n (%) | 318 (12.6%) | 74 (11.7%) | 85 (13.5%) | 85 (13.5%) | 74 (11.8%) | 0.632 |
| Dialysis duration, years | 3.5 (4.4) | 4.2 (4.9) | 3.5 (4.5) | 3.6 (4.3) | 2.8 (3.8) | <0.001 |
| Dialysis modality, n (%) |  |  |  |  |  | 0.022 |
| Haemodialysis | 2014 (80.0%) | 527 (83.7%) | 505 (80.3%) | 500 (79.4%) | 482 (76.8%) |  |
| Peritoneal dialysis | 503 (20.0%) | 103 (16.3%) | 124 (19.7%) | 130 (20.6%) | 146 (23.2%) |  |
| Diabetes, n (%) | 772 (30.7%) | 148 (23.5%) | 184 (29.3%) | 201 (31.9%) | 239 (38.1%) | <0.001 |
| Hypertension, n (%) | 2167 (86.1%) | 522 (82.9%) | 529 (84.1%) | 546 (86.7%) | 570 (90.8%) | <0.001 |
| Coronary heart disease, n (%) | 310 (12.3%) | 82 (13.0%) | 79 (12.6%) | 77 (12.2%) | 72 (11.5%) | 0.862 |
| Hyperlipidemia, n (%) | 434 (17.2%) | 90 (14.3%) | 108 (17.2%) | 112 (17.8%) | 124 (19.7%) | 0.080 |
| Stroke, n (%) | 364 (14.5%) | 100 (15.9%) | 89 (14.1%) | 85 (13.5%) | 90 (14.3%) | 0.670 |
| **Medication history, n (%)** |  |  |  |  |  |  |
| Vitamin D | 1252 (49.7%) | 290 (46.0%) | 321 (51.0%) | 294 (46.7%) | 347 (55.3%) | 0.003 |
| Calcium supplements | 680 (27.0%) | 160 (25.4%) | 156 (24.8%) | 176 (27.9%) | 188 (29.9%) | 0.145 |
| Cinacalcet | 365 (14.5%) | 86 (13.7%) | 91 (14.1%) | 89 (14.1%) | 99 (15.8%) | 0.742 |
| Non-calcium−containing phosphate binders | 1083 (43.0%) | 244 (38.7%) | 270 (42.9%) | 262 (41.6%) | 307 (48.9%) | 0.003 |
| **Laboratory results** |  |  |  |  |  |  |
| WBC, *10^9^/L | 6.2 (4.9−7.8) | 6.1 (4.8−7.7) | 5.9 (4.8−7.7) | 6.1 (4.9−7.7) | 6.5 (5.3−8.0) | 0.001 |
| Haemoglobin, g/L | 97.5 (22.4) | 100.3 (22.7) | 98.6 (21.6) | 97.7 (22.4) | 93.4 (22.2) | <0.001 |
| Albumin, g/L | 34.9 (5.6) | 34.6 (5.8) | 35.3 (5.3) | 35.1 (5.5) | 34.8 (6.0) | 0.107 |
| FPG, mmol/L | 5.2 (4.4−6.9) | 4.9 (4.2−6.4) | 5.2 (4.4−6.7) | 5.2 (4.4−6.9) | 5.5 (4.5−7.6) | <0.001 |
| Uric acid, μmol/L | 393.9 (132.1) | 386.0 (136.7) | 389.1 (129.6) | 400.2 (130.2) | 400.4 (131.6) | 0.111 |
| Triglycerides, mmol/L | 1.4 (1.0−2.0) | 1.3 (0.9−1.8) | 1.3 (1.0−1.9) | 1.4 (1.0−2.1) | 1.5 (1.1−2.3) | <0.001 |
| Total cholesterol, mmol/L | 3.8 (1.2) | 3.7 (1.3) | 3.8 (1.1) | 3.9 (1.2) | 3.9 (1.2) | 0.028 |
| HDL cholesterol, mmol/L | 1.0 (0.3) | 1.0 (0.3) | 1.0 (0.3) | 1.0 (0.3) | 0.9 (0.3) | 0.001 |
| LDL cholesterol, mmol/L | 2.2 (0.9) | 2.1 (0.9) | 2.1 (0.8) | 2.2 (0.9) | 2.2 (0.8) | 0.016 |
| AST, U/L | 15 (11 −20) | 16.0 (12.0−21.1) | 15.0 (11.0−19.5) | 14.8 (11.0−19.7) | 14.3 (11.0−20.0) | 0.006 |
| ALT, U/L | 11.0 (7.2−17.5) | 10.1 (7.0−17.0) | 11.0 (7.4−17.0) | 11.0 (7.2−18.0) | 11.3 (8.0−18.3) | 0.124 |
| GGT, U/L | 21 (14−35) | 22.2 (14.3−40.0) | 20.4 (14.0−33.2) | 20.0 (14.0−32.1) | 22.0 (14.5−36).1 | 0.027 |
| Bicarbonate, mmol/L | 22.5 (4.3) | 22.9 (4.2) | 22.6 (4.2) | 22.5 (4.1) | 22.2 (4.5) | 0.017 |
| Corrected serum calcium, mmol/L | 2.3 (0.2) | 2.3 (0.3) | 2.3 (0.2) | 2.3 (0.2) | 2.2 (0.2) | <0.001 |
| Serum Phosphate, mmol/L | 1.8 (0.6) | 1.7 (0.6) | 1.8 (0.6) | 1.9 (0.6) | 1.9 (0.7) | <0.001 |
| iPTH, pg/mL | 241.4 (123.6−437.0) | 210.3 (106.3−395.2) | 231.8 (129.2−440.1) | 261.1 (120.9−444.1) | 265.5 (137.0−456.1) | 0.014 |
| **CT data** |  |  |  |  |  |  |
| Log (TAC+1) | 4.4 ( 3.4) | 5.0 (3.3) | 4.6 (3.3) | 4.1 (3.4) | 4.0 (3.4) | <0.001 |
| Log (ATAC +1) | 0.5 (1.4) | 0.6 (1.6) | 0.6 (1.5) | 0.4 (1.3) | 0.4 (1.3) | 0.022 |
| Log (AoAC +1) | 3.8 (3.3) | 4.4 (3.2) | 4.0 (3.3) | 3.6 (3.3) | 3.4 (3.3) | <0.001 |
| Log (DTAC +1) | 3.0 (3.1) | 3.6 (3.2) | 3.1 (3.1) | 2.8 (3.1) | 2.6 (3.1) | <0.001 |

Data are presented as mean (SD), n (%), or median (IQR). Characteristics were compared using one-way ANOVA or Kruskal-Wallis for continuous variables, and χ2 for categorical variables. *P*< 0.05 was considered statistically significant. SMI indicates skeletal muscle index; BP, blood pressure; BMI, body mass index; WBC, white blood cell count; FPG: fasting plasma glucose; HDL, high−density lipoprotein; LDL, low−density lipoprotein; AST, aspartate aminotransferase; ALT, alanine aminotransferase; GGT, gamma−glutamyl transferase; iPTH, intact parathyroid hormone; TAC, thoracic aortic calcification; ATAC, ascending thoracic aortic calcification; AoAC, aortic arch calcification; DTAC, descending thoracic aortic calcification.

**Table S2. Regression Coefficients (β) for Association of SMI with TAC**

|  | **SMl quartile separated by sex** | | | |  |  |  |
| --- | --- | --- | --- | --- | --- | --- | --- |
|  | **Q1**  **(lowest)** | **Q2** | **Q3** | **Q4**  **(highest)** | ***P* for trend** | **Continuous variable**  **(Per 1 SD Increase in SMI)** | ***P* value** |
| **TAC** |  |  |  |  |  |  |  |
| Model 1 | 0(ref.) | −0.27 (−0.57 – 0.02) | −0.49 (−0.79 – −0.20) | −0.44 (−0.73 – −0.14) | 0.001 | −0.05 (−0.09 – −0.02) | 0.002 |
| Model 2 | 0(ref.) | −0.22 (−0.51 – 0.06) | −0.53 (−0.83 – −0.24) | −0.43 (−0.76 – −0.10) | 0.003 | −0.05 (−0.09 – −0.01) | 0.007 |
| Model 3 | 0(ref.) | −0.24 (−0.51 – 0.04) | −0.52 (−0.81 – −0.23) | −0.42 (−0.74 – −0.10) | 0.004 | −0.05 (−0.09 – −0.01) | 0.011 |
| **ATAC** |  |  |  |  |  |  |  |
| Model 1 | 0(ref.) | −0.03 (−0.18 – 0.13) | −0.11 (−0.26 – 0.05) | −0.12 (−0.28 – 0.03) | 0.081 | −0.04 (−0.09 – 0.00) | 0.046 |
| Model 2 | 0(ref.) | −0.01 (−0.16 – 0.15) | −0.10 (−0.27 – 0.06) | −0.10 (−0.28 – 0.08) | 0.199 | −0.04 (−0.09 – 0.01) | 0.134 |
| Model 3 | 0(ref.) | −0.01 (−0.16 – 0.15) | −0.09 (−0.26 – 0.07) | −0.10 (−0.28 – 0.09) | 0.228 | −0.03 (−0.08 – 0.02) | 0.190 |
| **AoAC** |  |  |  |  |  |  |  |
| Model 1 | 0(ref.) | −0.32 (−0.61 – −0.03) | −0.44 (−0.74 – −0.15) | −0.47 (−0.77 – −0.18) | 0.001 | −0.05 (−0.08 – −0.01) | 0.006 |
| Model 2 | 0(ref.) | −0.29 (−0.58 – −0.01) | −0.51 (−0.81 – −0.21) | −0.51 (−0.84 – −0.18) | 0.001 | −0.05 (−0.09 – −0.01) | 0.013 |
| Model 3 | 0(ref.) | −0.31 (−0.60 – −0.03) | −0.50 (−0.79 – −0.20) | −0.50 (−0.83 – −0.17) | 0.001 | −0.05 (−0.09 – −0.01) | 0.017 |
| **DTAC** |  |  |  |  |  |  |  |
| Model 1 | 0(ref.) | −0.41 (−0.70 – −0.12) | −0.55 (−0.84 – −0.26) | −0.52 (−0.81 – −0.22) | <0.001 | −0.07 (−0.11 – −0.03) | <0.001 |
| Model 2 | 0(ref.) | −0.31 (−0.59 – −0.02) | −0.48 (−0.77 – −0.18) | −0.34 (−0.67 – −0.01) | 0.024 | −0.05 (−0.09 – −0.01) | 0.020 |
| Model 3 | 0(ref.) | −0.32 (−0.60 – −0.04) | −0.46 (−0.75 – −0.17) | −0.32 (−0.65 – 0.00) | 0.031 | −0.05 (−0.09 – −0.01) | 0.031 |

Model 1: adjusted for SMD, age, and sex. Model 2: adjusted for all the covariates included in model 1 and additionally adjusted for BMI, smoking history, dialysis duration, history of hypertension, history of diabetes. Model 3: included all the covariates from model 2 and additionally adjusted for log WBC, log (TG + 1), LDL cholesterol, log iPTH, serum phosphate, corrected serum calcium, and Vitamin D use.

SMI indicates skeletal muscle index; SMD, skeletal muscle radiodensity; SD, standard deviation; BMI, body mass index; WBC, white blood cell count; TG, triglycerides; LDL, low-density lipoprotein; iPTH, intact parathyroid hormone; TAC, thoracic aortic calcification; ATAC, ascending thoracic aortic calcification; AoAC, aortic arch calcification; DTAC, descending thoracic aortic calcification.

**Table S3. Baseline Characteristics of Subjects by TAC Progression**

|  | Over all | Slow Progressors (*n* = 272) | Rapid Progressors (*n* = 272) | *P* value |
| --- | --- | --- | --- | --- |
| Annualized absolute rate of change in TAC | 97.0 (1.5−600.8) | 1.5 (0.0−25.5) | 598.1 (255.1−1198.7) |  |
| Age, years | 51.7 (13.3) | 45.0 (12.4) | 58.6 (10.4) | <0.001 |
| Systolic BP, mm Hg | 152.6 (25.9) | 152.8 (27.2) | 152.5 (24.7) | 0.863 |
| Diastolic BP, mm Hg | 88.2 (16.8) | 90.7 (17.8) | 85.7 (15.3) | <0.001 |
| BMI, kg/m^2^ | 23.6 (3.8) | 23.2 (3.7) | 24.1 (3.9) | 0.012 |
| Smoking history, n (%) | 177 (32.5%) | 47 (17.3%) | 76 (27.9%) | 0.003 |
| Dialysis modality, n (%) |  |  |  | <0.001 |
| Haemodialysis | 367 (67.5%) | 165 (60.7%) | 202 (74.3%) |  |
| Peritoneal dialysis | 177 (32.5%) | 107 (39.3%) | 70 (25.7%) |  |
| Diabetes, n (%) | 198 (36.4%) | 78 (28.7%) | 120 (44.1%) | <0.001 |
| Hypertension, n (%) | 508 (93.4%) | 249 (91.5%) | 259 (95.2%) | 0.085 |
| Coronary heart disease, n (%) | 54 (9.9%) | 11 (4%) | 43 (15.8%) | <0.001 |
| Hyperlipidemia, n (%) | 53 (9.7%) | 30 (11%) | 23 (8.5%) | 0.311 |
| Stroke, n (%) | 48 (8.8%) | 13 (4.8%) | 35 (12.9%) | <0.001 |
| **Medication history, n (%)** |  |  |  |  |
| Vitamin D | 282 (51.8%) | 148 (54.4%) | 134 (49.3%) | 0.230 |
| Calcium supplements | 219 (40.3%) | 111 (40.8%) | 108 (39.7%) | 0.793 |
| Cinacalcet | 9 (1.7%) | 4 (1.5%) | 5 (1.8%) | 0.737 |
| Non-calcium−containing phosphate binders | 53 (9.7%) | 30 (11%) | 23 (8.5%) | 0.311 |
| **Laboratory results** |  |  |  |  |
| WBC, *10^9^/L | 6.3 (5.3−8.0) | 6.1 (5.0−7.7) | 6.5 (5.4−8.2) | 0.017 |
| Haemoglobin, g/L | 83.4 (16.7) | 81.9 (16.1) | 84.9 (17.2) | 0.036 |
| Albumin, g/L | 33.3 (5.3) | 33.7 (5.4) | 32.9 (5.3) | 0.098 |
| FPG, mmol/L | 5.2 (4.6−6.6) | 5.0 (4.4−6.2) | 5.5 (4.8−7.1) | <0.001 |
| Uric acid, μmol/L | 471.4 (146.7) | 476.3 (153.5) | 466.5 (139.7) | 0.436 |
| Triglycerides, mmol/L | 1.5 (1.1−2.0) | 1.5 (1.1−2.0) | 1.5 (1.1−1.9) | 0.619 |
| Total cholesterol, mmol/L | 4.2 (1.2) | 4.2 (1.2) | 4.3 (1.2) | 0.354 |
| HDL cholesterol, mmol/L | 1.0 (0.3) | 1.0 (0.3) | 1.0 (0.3) | 0.949 |
| LDL cholesterol, mmol/L | 2.4 (0.9) | 2.4 (0.9) | 2.4 (0.9) | 0.354 |
| AST, U/L | 16.0 (12.0−22.0) | 15.0 (12.0−22.6) | 16.6 (12.0−22.0) | 0.617 |
| ALT, U/L | 14.0 (10.0−22.0) | 14.5 (9.2−22.5) | 14.0 (10.0−21.4) | 0.794 |
| GGT, U/L | 26.1 (16.0−48.0) | 23.0 (14.9−44.0) | 28.0 (17.0−51.5) | 0.018 |
| Bicarbonate, mmol/L | 21.4 (4.6) | 21.4 (4.6) | 21.5 (4.6) | 0.850 |
| Corrected serum calcium, mmol/L | 2.3 (0.3) | 2.3 (0.3) | 2.3 (0.3) | 0.705 |
| Serum Phosphate, mmol/L | 1.8 (0.6) | 1.8 (0.6) | 1.7 (0.6) | 0.184 |
| iPTH, pg/mL | 213.3 (107.7−370.1) | 231.1 (123.7−392.9) | 183.4 (96.3−349.1) | 0.019 |
| **CT data** |  |  |  |  |
| SMD, HU | 37.6 (8.1) | 40.4 (7.1) | 34.9 (8.3) | <0.001 |
| SMI, cm^2^/m^2^ | 38.5 (8.7) | 38.9 (8.6) | 38.5 (8.8) | 0.549 |
| Baseline TAC, mm³ | 0.0 (0.0−168.6) | 0.0 (0.0−118.8) | 0.3 (0.0−234.2) | 0.128 |

Data are presented as mean (SD), n (%), or median (IQR). Characteristics were compared using t-test or Kruskal-Wallis tests for continuous variables, and χ2 tests for categorical variables. TAC indicates thoracic aortic calcification; BP, blood pressure; BMI, body mass index; WBC, white blood cell count; FPG: fasting plasma glucose; HDL, high−density lipoprotein; LDL, low−density lipoprotein; AST, aspartate aminotransferase; ALT, alanine aminotransferase; GGT, gamma−glutamyl transferase; iPTH, intact parathyroid hormone; SMD, skeletal muscle radiodensity; SMI, skeletal muscle index.

**Table S4. Regression Coefficients (β) for Association of SMI with TAC Progression**

|  | **SMl quartile separated by sex** | | | |  |  |  |
| --- | --- | --- | --- | --- | --- | --- | --- |
|  | **Q1**  **(lowest)** | **Q2** | **Q3** | **Q4**  **(highest)** | ***P* for trend** | **Continuous variable**  **(Per 1 SD Increase in SMI)** | ***P* value** |
| **TAC** |  |  |  |  |  |  |  |
| Model 1 | 0(ref.) | −0.24 (−0.77 – 0.28) | −0.72 (−1.25 – −0.20) | −0.23 (−0.77 – 0.31) | 0.190 | −0.06 (−0.14 – 0.01) | 0.100 |
| Model 2 | 0(ref.) | −0.30 (−0.83 – 0.22) | −0.92 (−1.48 – −0.37) | −0.59 (−1.21 – 0.04) | 0.019 | −0.13 (−0.21 – −0.04) | 0.005 |
| Model 3 | 0(ref.) | −0.19 (−0.65 – 0.28) | −0.71 (−1.20 – −0.23) | −0.58 (−1.13 – −0.03) | 0.013 | −0.12 (−0.20 – −0.04) | 0.003 |
| **ATAC** |  |  |  |  |  |  |  |
| Model 1 | 0(ref.) | −0.14 (−0.43 – 0.16) | −0.01 (−0.31 – 0.28) | −0.07 (−0.38 – 0.24) | 0.808 | −0.01 (−0.10 – 0.08) | 0.831 |
| Model 2 | 0(ref.) | −0.16 (−0.46 – 0.14) | −0.08 (−0.39 – 0.24) | −0.20 (−0.55 – 0.16) | 0.337 | −0.05 (−0.16 – 0.05) | 0.326 |
| Model 3 | 0(ref.) | −0.22 (−0.49 – 0.06) | −0.19 (−0.48 – 0.10) | −0.31 (−0.63 – 0.02) | 0.080 | −0.08 (−0.18 – 0.02) | 0.108 |
| **AoAC** |  |  |  |  |  |  |  |
| Model 1 | 0(ref.) | −0.18 (−0.69 – 0.33) | −0.61 (−1.12 – −0.10) | 0.10 (−0.43 – 0.63) | 0.898 | −0.02 (−0.10 – 0.05) | 0.583 |
| Model 2 | 0(ref.) | −0.22 (−0.73 – 0.30) | −0.72 (−1.26 – −0.18) | −0.11 (−0.72 – 0.50) | 0.389 | −0.06 (−0.15 – 0.02) | 0.157 |
| Model 3 | 0(ref.) | −0.10 (−0.56 – 0.36) | −0.45 (−0.93 – 0.03) | −0.14 (−0.69 – 0.40) | 0.380 | −0.06 (−0.14 – 0.02) | 0.150 |
| **DTAC** |  |  |  |  |  |  |  |
| Model 1 | 0(ref.) | −0.14 (−0.66 – 0.38) | −0.74 (−1.26 – −0.22) | −0.44 (−0.98 – 0.10) | 0.032 | −0.10 (−0.18 – −0.03) | 0.008 |
| Model 2 | 0(ref.) | −0.16 (−0.69 – 0.37) | −0.84 (−1.39 – −0.29) | −0.59 (−1.21 – 0.03) | 0.018 | −0.14 (−0.23 – −0.05) | 0.003 |
| Model 3 | 0(ref.) | −0.23 (−0.69 – 0.23) | −0.86 (−1.34 – −0.37) | −0.55 (−1.09 – 0.00) | 0.012 | −0.12 (−0.20 – −0.04) | 0.004 |

Model 1: adjusted for SMD, age, and sex. Model 2: adjusted for all the covariates included in model 1 and additionally adjusted for BMI, smoking history, history of hypertension, history of diabetes. Model 3: included all the covariates from model 2 and additionally adjusted for log WBC, log (TG + 1), LDL cholesterol, log iPTH, serum phosphate, corrected serum calcium, Vitamin D use, and log (baseline calcification score + 1).

SMI indicates skeletal muscle index; SMD, skeletal muscle radiodensity; SD, standard deviation;

BMI, body mass index; WBC, white blood cell count; TG, triglycerides; LDL, low-density lipoprotein; iPTH, intact parathyroid hormone; TAC, thoracic aortic calcification; ATAC, ascending thoracic aortic calcification; AoAC, aortic arch calcification; DTAC, descending thoracic aortic calcification.

**Table S5.** **Regression Coefficients (β) for Association of SMD with TAC Progression after Excluding Patients Diagnosed with Stroke**

|  | **SMD quartile separated by sex** | | | |  |  |  |
| --- | --- | --- | --- | --- | --- | --- | --- |
|  | **Q1**  **(lowest)** | **Q2** | **Q3** | **Q4**  **(highest)** | ***P* for trend** | **Continuous variable**  **(Per 1 SD Increase in SMD)** | ***P* value** |
| **TAC** |  |  |  |  |  |  |  |
| Model 1 | 0(ref.) | −0.87 (−1.46 – −0.29) | −1.13 (−1.73 – −0.54) | −1.20 (−1.85 – −0.55) | <0.001 | −0.14 (−0.23 – −0.06) | 0.001 |
| Model 2 | 0(ref.) | −0.82 (−1.41 – −0.24) | −0.99 (−1.59 – −0.38) | −1.16 (−1.81 – −0.51) | 0.001 | −0.13 (−0.22 – −0.04) | 0.003 |
| Model 3 | 0(ref.) | −0.66 (−1.19 – −0.14) | −0.68 (−1.22 – −0.13) | −0.95 (−1.53 – −0.36) | 0.003 | −0.11 (−0.19 – −0.03) | 0.006 |

Model 1: adjusted for SMI, age, and sex. Model 2: adjusted for all the covariates included in model 1 and additionally adjusted for BMI, smoking history, history of hypertension, history of diabetes. Model 3: included all the covariates from model 2 and additionally adjusted for log WBC, log (TG + 1), LDL cholesterol, log iPTH, serum phosphate, corrected serum calcium, Vitamin D use, and log (baseline TAC + 1).

SMI indicates skeletal muscle index; SMD, skeletal muscle radiodensity; SD, standard deviation;

BMI, body mass index; WBC, white blood cell count; TG, triglycerides; LDL, low−density lipoprotein; iPTH, intact parathyroid hormone; TAC, thoracic aortic calcification.

**Table S6.** **Regression Coefficients (β) for Association of SMI with TAC Progression after Excluding Patients Diagnosed with Stroke**

|  | **SMI quartile separated by sex** | | | |  |  |  |
| --- | --- | --- | --- | --- | --- | --- | --- |
|  | **Q1**  **(lowest)** | **Q2** | **Q3** | **Q4**  **(highest)** | ***P* for trend** | **Continuous variable**  **(Per 1 SD Increase in SMI)** | ***P* value** |
| **TAC** |  |  |  |  |  |  |  |
| Model 1 | 0(ref.) | −0.10 (−0.66 – 0.45) | −0.63 (−1.19 – −0.07) | −0.20 (−0.77 – 0.37) | 0.254 | −0.07 (−0.15 – 0.01) | 0.088 |
| Model 2 | 0(ref.) | −0.16 (−0.71 – 0.40) | −0.84 (−1.43 – −0.26) | −0.58 (−1.23 – 0.07) | 0.032 | −0.14 (−0.23 – −0.04) | 0.004 |
| Model 3 | 0(ref.) | −0.04 (−0.53 – 0.45) | −0.65 (−1.17 – −0.13) | −0.62 (−1.20 – −0.04) | 0.015 | −0.14 (−0.22 – −0.05) | 0.001 |

Model 1: adjusted for SMD, age, and sex. Model 2: adjusted for all the covariates included in model 1 and additionally adjusted for BMI, smoking history, history of hypertension, history of diabetes. Model 3: included all the covariates from model 2 and additionally adjusted for log WBC, log (TG + 1), LDL cholesterol, log iPTH, serum phosphate, corrected serum calcium, Vitamin D use, and log (baseline TAC + 1).

SMI indicates skeletal muscle index; SMD, skeletal muscle radiodensity; SD, standard deviation;

BMI, body mass index; WBC, white blood cell count; TG, triglycerides; LDL, low−density lipoprotein; iPTH, intact parathyroid hormone; TAC, thoracic aortic calcification.

**Table S7. Regression Coefficients (β) for Association of SMD with TAC Progression after Excluding Patients Diagnosed with Stroke or Coronary Heart Disease**

|  | **SMD quartile separated by sex** | | | |  |  |  |
| --- | --- | --- | --- | --- | --- | --- | --- |
|  | **Q1**  **(lowest)** | **Q2** | **Q3** | **Q4**  **(highest)** | ***P* for trend** | **Continuous variable**  **(Per 1 SD Increase in SMD)** | ***P* value** |
| **TAC** |  |  |  |  |  |  |  |
| Model 1 | 0(ref.) | −1.02 (−1.65 – −0.38) | −1.22 (−1.86 – −0.59) | −1.24 (−1.93 – −0.55) | 0.001 | −0.14 (−0.24 – −0.05) | 0.002 |
| Model 2 | 0(ref.) | −0.95 (−1.58 – −0.31) | −1.04 (−1.69 – −0.39) | −1.16 (−1.85 – −0.47) | 0.002 | −0.13 (−0.22 – −0.04) | 0.005 |
| Model 3 | 0(ref.) | −0.79 (−1.36 – −0.21) | −0.80 (−1.38 – −0.21) | −1.00 (−1.63 – −0.38) | 0.004 | −0.11 (−0.20 – −0.03) | 0.007 |

Model 1: adjusted for SMI, age, and sex. Model 2: adjusted for all the covariates included in model 1 and additionally adjusted for BMI, smoking history, history of hypertension, history of diabetes. Model 3: included all the covariates from model 2 and additionally adjusted for log WBC, log (TG + 1), LDL cholesterol, log iPTH, serum phosphate, corrected serum calcium, Vitamin D use, and log (baseline TAC + 1).

SMI indicates skeletal muscle index; SMD, skeletal muscle radiodensity; SD, standard deviation;

BMI, body mass index; WBC, white blood cell count; TG, triglycerides; LDL, low−density lipoprotein; iPTH, intact parathyroid hormone; TAC, thoracic aortic calcification.

**Table S8. Regression Coefficients (β) for Association of SMI with TAC Progression after Excluding Patients Diagnosed with Stroke or Coronary Heart Disease**

|  | **SMl quartile separated by sex** | | | |  |  |  |
| --- | --- | --- | --- | --- | --- | --- | --- |
|  | **Q1**  **(lowest)** | **Q2** | **Q3** | **Q4**  **(highest)** | ***P* for trend** | **Continuous variable**  **(Per 1 SD Increase in SMI)** | ***P* value** |
| **TAC** |  |  |  |  |  |  |  |
| Model 1 | 0(ref.) | −0.12 (−0.69 – 0.46) | −0.71 (−1.30 – −0.12) | −0.28 (−0.88 – 0.32) | 0.159 | −0.08 (−0.16 – 0.01) | 0.077 |
| Model 2 | 0(ref.) | −0.18 (−0.75 – 0.40) | −0.99 (−1.60 – −0.37) | −0.73 (−1.42 – −0.05) | 0.011 | −0.15 (−0.25 – −0.06) | 0.002 |
| Model 3 | 0(ref.) | −0.05 (−0.57 – 0.46) | −0.74 (−1.29 – −0.18) | −0.75 (−1.36 – −0.13) | 0.006 | −0.15 (−0.24 – −0.06) | 0.001 |

Model 1: adjusted for SMD, age, and sex. Model 2: adjusted for all the covariates included in model 1 and additionally adjusted for BMI, smoking history, history of hypertension, history of diabetes. Model 3: included all the covariates from model 2 and additionally adjusted for log WBC, log (TG + 1), LDL cholesterol, log iPTH, serum phosphate, corrected serum calcium, Vitamin D use, and log (baseline TAC + 1).

SMI indicates skeletal muscle index; SMD, skeletal muscle radiodensity; SD, standard deviation;

BMI, body mass index; WBC, white blood cell count; TG, triglycerides; LDL, low−density lipoprotein; iPTH, intact parathyroid hormone; TAC, thoracic aortic calcification.
